# Zero-Mode Waveguide Nanowells for Single-Molecule Detection in Living Cells

**DOI:** 10.1021/acsnano.3c05959

**Published:** 2023-10-04

**Authors:** Sora Yang, Nils Klughammer, Anders Barth, Marvin E. Tanenbaum, Cees Dekker

**Affiliations:** † Oncode Institute, Hubrecht Institute−KNAW and University Medical Center Utrecht, Uppsalalaan 8, 3584 CT, Utrecht, The Netherlands; ‡ Department of Bionanoscience, Kavli Institute of Nanoscience, Delft University of Technology, Van der Maasweg 9, 2629 HZ, Delft, The Netherlands

**Keywords:** single-molecule fluorescence, zero-mode waveguide, fluorescence enhancement, live-cell imaging, fluorescence microscopy, palladium, fluorescence correlation spectroscopy

## Abstract

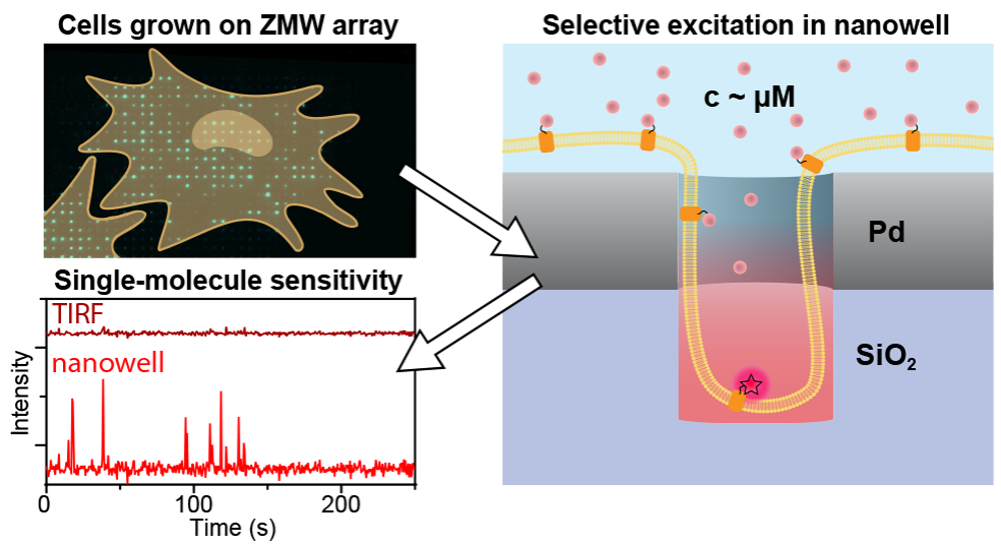

Single-molecule fluorescence imaging experiments generally require sub-nanomolar protein concentrations to isolate single protein molecules, which makes such experiments challenging in live cells due to high intracellular protein concentrations. Here, we show that single-molecule observations can be achieved in live cells through a drastic reduction in the observation volume using overmilled zero-mode waveguides (ZMWs- subwavelength-size holes in a metal film). Overmilling of the ZMW in a palladium film creates a nanowell of tunable size in the glass layer below the aperture, which cells can penetrate. We present a thorough theoretical and experimental characterization of the optical properties of these nanowells over a wide range of ZMW diameters and overmilling depths, showing an excellent signal confinement and a 5-fold fluorescence enhancement of fluorescent molecules inside nanowells. ZMW nanowells facilitate live-cell imaging as cells form stable protrusions into the nanowells. Importantly, the nanowells greatly reduce the cytoplasmic background fluorescence, enabling the detection of individual membrane-bound fluorophores in the presence of high cytoplasmic expression levels, which could not be achieved with TIRF microscopy. Zero-mode waveguide nanowells thus provide great potential to study individual proteins in living cells.

## Introduction

Single-molecule techniques are widely applied to study the behavior of biomolecules or biomolecular complexes, providing mechanistic insights into individual steps of biological processes that would otherwise be averaged out in bulk experiments.^1^ Imaging-based approaches have been especially powerful in studying single nucleic acid and protein molecules, as they allow tracking of individual biomolecules in space and time. Central to all single-molecule fluorescence imaging techniques is the ability to detect and distinguish a single molecule of interest over the background of fluorescent molecules that are freely diffusing through the solution. The ability to isolate a single molecule by imaging therefore depends on the concentrations of fluorescent molecules and the observation volume; if multiple freely diffusing molecules are present within the observation volume, the isolation of one specific molecule of interest becomes very challenging.^2^

In *in vitro* experiments, single-molecule observation can easily be achieved by using low concentrations of fluorescent molecules, which limits the number of molecules in the observation volume. However, weak biomolecular interactions (*K*_d_ > 1 μM) that require high concentrations cannot be studied at the nano- to picomolar concentrations that are typically employed in *in vitro* single-molecule experiments. Moreover, studying biomolecules in their natural habitat, the crowded environment of live cells, is also very challenging, as protein concentrations in cells are often in the high nanomolar to micromolar range,^3^ which is incompatible with single-molecule observations.^2^ Fundamentally, the concentration limit for single-molecule observation is bounded by the size of the observation volume, which can be minimized using common optical sectioning methods such as confocal microscopy, total internal reflection microscopy (TIRF), or light-sheet microscopy.^4^ Despite such improvements, the volumes remain on the order of femtoliters, which puts the concentration limit for isolating single molecules at ≈1 nM.^2,5^

A much more drastic confinement of the observation volume can be achieved using zero-mode waveguides (ZMWs), which are subwavelength apertures in a metal film. Owing to their small size (≈100 nm), ZMWs effectively block the propagation of incident light of wavelengths above a characteristic cutoff wavelength λ_c_, λ > λ_c_ = 1.7*d*, where *d* is the diameter of the aperture. Within the ZMW, an evanescent field forms which to first order follows an exponential decay as .^6^ Typical decay lengths are on the scale of several tens to hundreds of nanometers, depending on the ZMW diameter, the wavelength of incident light in the surrounding medium, and the ZMW material. Thus, by providing observation volumes in the zeptoliter range, ZMWs enable single-molecule studies at even micromolar concentrations.^7^ ZMWs made from gold or aluminum have been extensively studied^7−14^ and used for a variety of *in vitro* single-molecule applications^15−21^ and notably for DNA sequencing.^22^ Recently, we have introduced the use of palladium for free-standing ZMWs,^23^ which were applied to the *in vitro* study of nucleocytoplasmic transport.^24^ Palladium offers excellent mechanical and chemical stability, can easily be modified *via* thiol chemistry,^24−26^ and provides reduced photoluminescence in the blue spectral region compared to gold.^23,27,28^ Importantly, Pd is compatible with live cell experiments due to its low cytotoxicity.^29^

**Figure 1:**
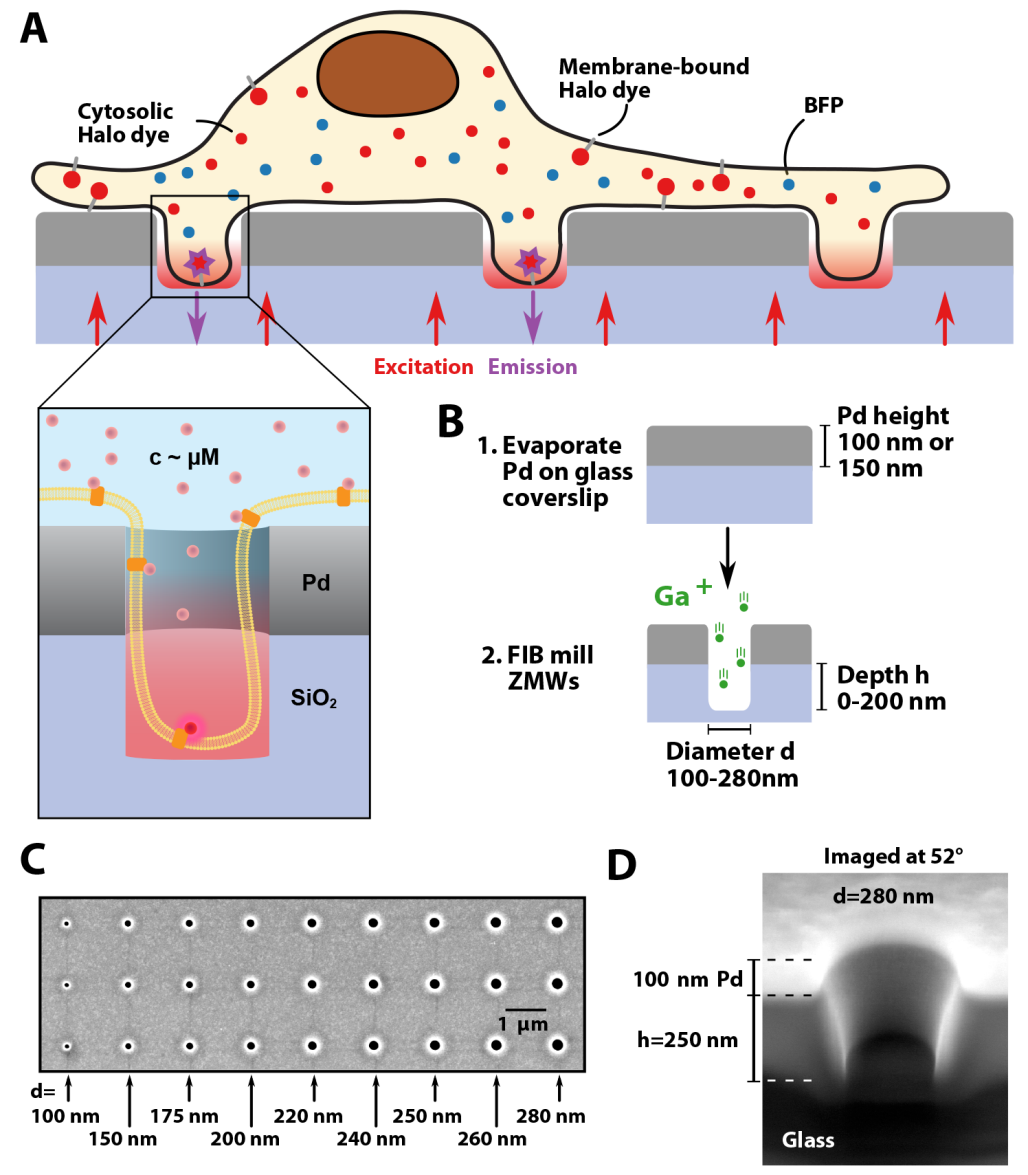
Schematic of the experiment and fabrication of overmilled ZMWs. (A) Schematic of a cell on top of an array of overmilled ZMWs. Nanowells below Pd ZMWs allow for the observation of single membrane bound fluorophores despite a high abundance of cytoplasmicfluorophores. (B) Pd is evaporated onto a glass coverslip, and ZWMs are created by focused ion beam milling. Pore diameters used in the study ranged between 100 nm and 280 nm, and overmilling depths ranged between 0 nm and 200 nm. (C) SEM image showing ZMWs with different pore diameters. (D) The depth of milling was measured by cutting through the pores with a focused ion beam and measuring the height when imaging under an angle of 52°.

While the vast majority of studies applying ZMWs to single-molecule measurements have been performed *in vitro*, a few studies have shown that ZMWs made of aluminum can be applied to single-molecule imaging of cellular (membrane) proteins as well, as cells can form protrusions that penetrate into ZMWs,^30−32^ which has enabled single-molecule observation of membrane composition^30^ and membrane channels.^32^ Inspired by this work, we hypothesized that the creation of nanowells in the glass coverslip below the ZMWs (see Figure 1A) provides a means of fine-tuning the size of the observation volume and results in excellent optical properties, while allowing cellular protrusions to enter the nanowells.^33,34^ Moving the observation volume slightly away from the ZMW cavity can potentially lead to an increase of the single-molecule fluorescence signal due to enhancement of the excitation field or modulation of the radiative and nonradiative rates by the metal, as has previously been shown for aluminum^35−37^ and gold.^12^ Overmilling could also allow cells to penetrate more deeply through the ZMWs and allow facile imaging not only of membrane-bound proteins but also of proteins in the cytoplasm, greatly expanding the potential applications of ZMW imaging of living cells.

Here, we establish palladium ZMW nanowells as a tool for single-molecule studies in live cells (Figure 1A). We fabricated ZMW arrays using focused ion beam (FIB) milling, which allowed us to survey a wide range of diameters and overmilling depths to optimize the design for both optical performance and cell compatibility. Finite-difference time-domain (FDTD) simulations of the excitation intensity and fluorescence emission showed an effective reduction of the observation volume to the nanowell below the ZMW and suggested a potential fluorescence enhancement due to the focusing of the excitation intensity within the well, facilitated by the formation of a standing wave below the metal layer. The theoretical results are corroborated by single-molecule experiments on freely diffusing fluorophores, which confirmed the signal confinement and showed an up to 5-fold fluorescence enhancement. Using live-cell imaging, we show that human osteosarcoma U2OS cells readily protruded into the nanowells, which occurred more efficiently when ZMWs were overmilled. Cell protrusions remained stable over the time scale of minutes, enabling single-molecule observation of individual membrane-bound fluorophores even in the presence of high cytoplasmic concentrations of the same fluorophores. This was only possible due to the efficient suppression of the cytoplasmic background signal by the ZMW, whereas conventional TIRF microscopy did not allow single molecules to be followed in this setting. Oblique illumination of the nanowells leads to a further reduction of the background levels. Due to their excellent cell compatibility, overmilled Pd ZMWs can be readily applied for single-molecule studies of biological processes in living cells at physiological concentrations.

## Results

### Fabrication of Pd ZMWs on Glass

To fabricate nanowells, we first applied a thin (100 or 150 nm) palladium layer to standard glass coverslips covered with a 5 nm Ti adhesion layer by physical vapor deposition (Figure 1B). In contrast to previous studies that used aluminum,^30−32^ we chose palladium due to its suitability for nanostructuring, good chemical stability, low photoluminescence in the visible spectrum, and low cytotoxicity.^23,26,29^ Palladium surfaces can also easily be functionalized using thiols, which provides a strategy for the specific immobilization of molecules and thus can be used for surface passivation *via* self-assembled monolayers or may be useful for promoting cell adhesion for certain cell types.^25^ As in our previous studies,^23,24^ we used focused ion beam milling to create pores in the metal layer, which allows the precise tuning of pore diameters and pore depths within a single array (Figure 1B). We manufactured arrays containing pores of different sizes and depths, including larger marker holes for identification of the different areas within the arrays (Supplementary Figure 1). Typically, 16 arrays were placed on a single glass coverslip, each containing ≈3000 nanowells of varying diameter and depth (Figure 1C, Supplementary Figure 1). Pore diameters were chosen to range between 100 nm and 280 nm based on a previous study that showed cell protrusion into ZMWs.^32^ The depth of the nanowells was varied by overmilling into the glass surface below the palladium layer up to 200 nm. An example cross-section is shown in Figure 1D.

### Simulating the Optical Properties of Palladium ZMW Nanowells

**Figure 2:**
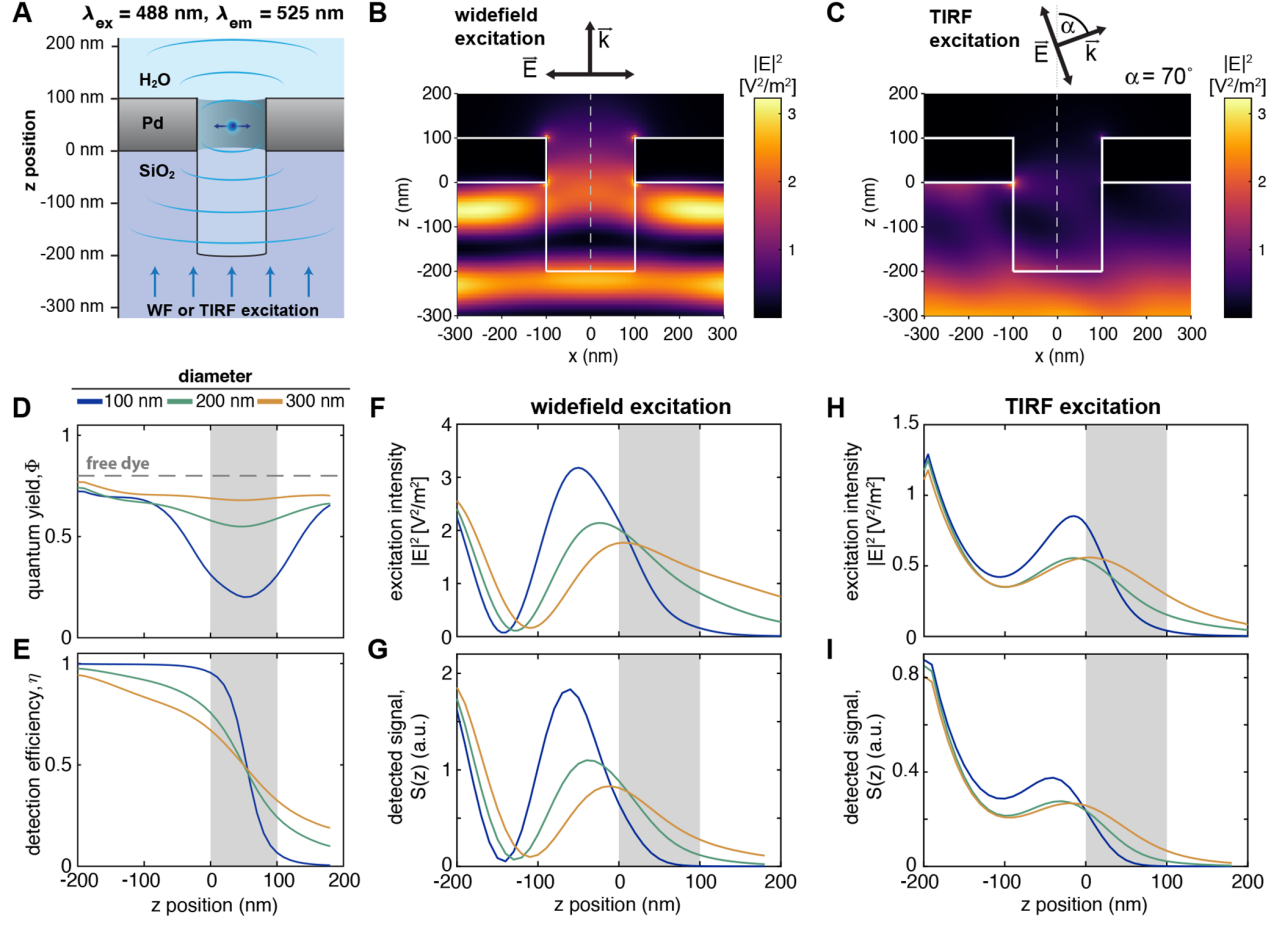
FDTD simulations of the excitation field and fluorescence emission within a nanowell underneath a ZMW. (A) Schematic of the simulation setup. A dipole was placed at varying depths within the aperture and excited by a plane wave incident from the bottom (wide-field, WF) or under an angle of 70° resembling conditions used in TIRF microscopy. (B, C) Resulting distributions of the excitation field intensity for wide-field (B) or TIRF (C) excitation for a ZMW diameter of 200 nm and an overmilling depth of 200 nm. The electric field is polarized along the *x*-axis. (D, E) Computed quantum yield and detection efficiency of the dye Alexa488 as a function of the *z*-position along the central pore axis (dashed line in B, C). (F–I) Z-profiles of the excitation intensity along the central pore axis (F, H) and the detected signal *S*(*z*) (G, I) under wide-field (F, G) and TIRF (H, I) excitation. The position of the metal membrane is indicated as a gray-shaded area.

To guide the selection of the optimal width and depth of the well below the ZMW, we performed FDTD simulations of the excitation electromagnetic field and dipole emission within overmilled ZMWs (Figure 2A). These simulations allow us to assess the spatial distribution of the excitation intensity, the modulation of the fluorescence quantum yield of the fluorophore, and the fraction of signal directed toward the detection side, which together define the detectable signal from within the nanowell as the product of these quantities.

To cover the different excitation modes applied in this study, we probed the excitation field distribution at wavelengths of 488 nm and 640 nm upon excitation by a plane wave (wide-field), under angled illumination as used in TIRF microscopy (Figure 2B,C,F,H, Supplementary Figures 2–5), as well as upon excitation by a focused beam as used in confocal microscopy (Supplementary Figures 6, 7). As expected, the zero-mode waveguide effectively blocks the propagation of the excitation light under all conditions for small pore diameters of 100 nm or below, as evident from the profiles of the excitation intensity along the pore axis (Figure 2F,H). At large pore diameters of 200 nm and above, a finite amount of excitation light propagates beyond the ZMW. Due to the reflective surface of the metal, a standing wave is formed on the detection side, which leads to an undulation of the excitation field intensity within the overmilled volume (Figure 2B).^12,36,37^ Under TIRF illumination at an angle of 70°, the first maximum of the standing wave pattern is shifted to longer distances from the metal surface compared to wide-field excitation because the magnitude of the wave vector orthogonal to the metal surface is reduced (Figure 2C). This results in a reduced excitation intensity within the well, but also provides a more even intensity distribution with an intensity maximum at the bottom of the well. Additionally, the propagation of light through the ZMW is reduced under TIRF illumination compared to wide-field excitation, which may limit background cytoplasmic fluorescence in imaging experiments (Figure 2H).

In addition to modulating the excitation field, the metal nanostructure affects the quantum yield of the fluorophore by modulating radiative and nonradiative decay rates, which we assess by simulating dipole emission at varying depths along the central axis of the nanowell (Figure 2A,D, Supplementary Figures 8, 9). Within the ZMW, the radiative rate is reduced while the nonradiative rate is strongly increased due to coupling to the metal nanostructure (gray area in Supplementary Figure 10). As the distance to the metal increases, the nonradiative losses decrease, while the radiative rate remains relatively constant within the volume beneath the ZMW. Overall, within the proximity of the ZMW, these effects lead to a strong predicted reduction of the quantum yield (Figure 2D) and hence the fluorescence lifetime (Supplementary Figures 8D, 9D), as will be assessed experimentally below. Within the nanowell below the ZMW, the modulation of the decay rates was only weakly dependent on the lateral position (Supplementary Figure 11). Finally, we consider the fraction of the fluorescence emission that can be detected in the experiment, *i*.*e*., the signal emitted toward the lower side of the ZMW facing the objective lens. Part of the dipole emission from within the ZMW is lost as it propagates toward the upper side of the ZMW that faces away from the objective lens, leading to a sharp decay of the detection efficiency within the ZMW (Figure 2E). Below the ZMW, the effective detection efficiency of the dipole emission is increased approximately 2-fold compared to the absence of a metal nanostructure because the metal layer acts as a mirror and propagation of radiation through the ZMW is blocked (Figure 2E, Supplementary Figures 8, 9). Overall, these processes lead to a more effective restriction of the detected signal to the well below the ZMW compared to what is expected from the excitation intensity alone.

The end result is a near-complete suppression of background signals originating from the top side of the ZMW. Under wide-field excitation, the simulations predict a background level of 3% at a pore diameter of 100 nm in a 100 nm thin Pd film, which increases to 10% at 300 nm diameter (numbers are given for an overmilling depth of 200 nm, Supplementary Figure 12A,D). Under TIRF excitation, the background level decreases further by approximately a factor of 2 compared to wide-field excitation because the excitation intensity is more effectively confined to the nanowell, reaching an excellent signal-to-background ratio of ∼25 even for a large pore diameter of 300 nm at an overmilling depth of 200 nm (Supplementary Figure 12E).

In summary, the FDTD simulations show that the observed signal remains effectively confined to the overmilled volume and the ZMW even for pore diameters of up to 300 nm (Figure 2 G,I, Supplementary Figures 8–12). Notably, no enhancement of the fluorescence emission is expected, as the presence of the metal waveguide is found to significantly reduce the fluorescence quantum yield in its immediate proximity (Figure 2D). On the other hand, the excitation field is enhanced within the nanowell due to the formation of the standing wave, reaching peak intensities that are up to three times higher compared to the absence of a waveguide (Figure 2B–H), and the detection efficiency is increased 2-fold because the dipole emission is directed toward the detection side (Figure 2E). Together, these effects lead to a significant predicted enhancement of the detected signal from the nanowells.

### Nanowells Provide Signal Confinement and Enhancement

**Figure 3:**
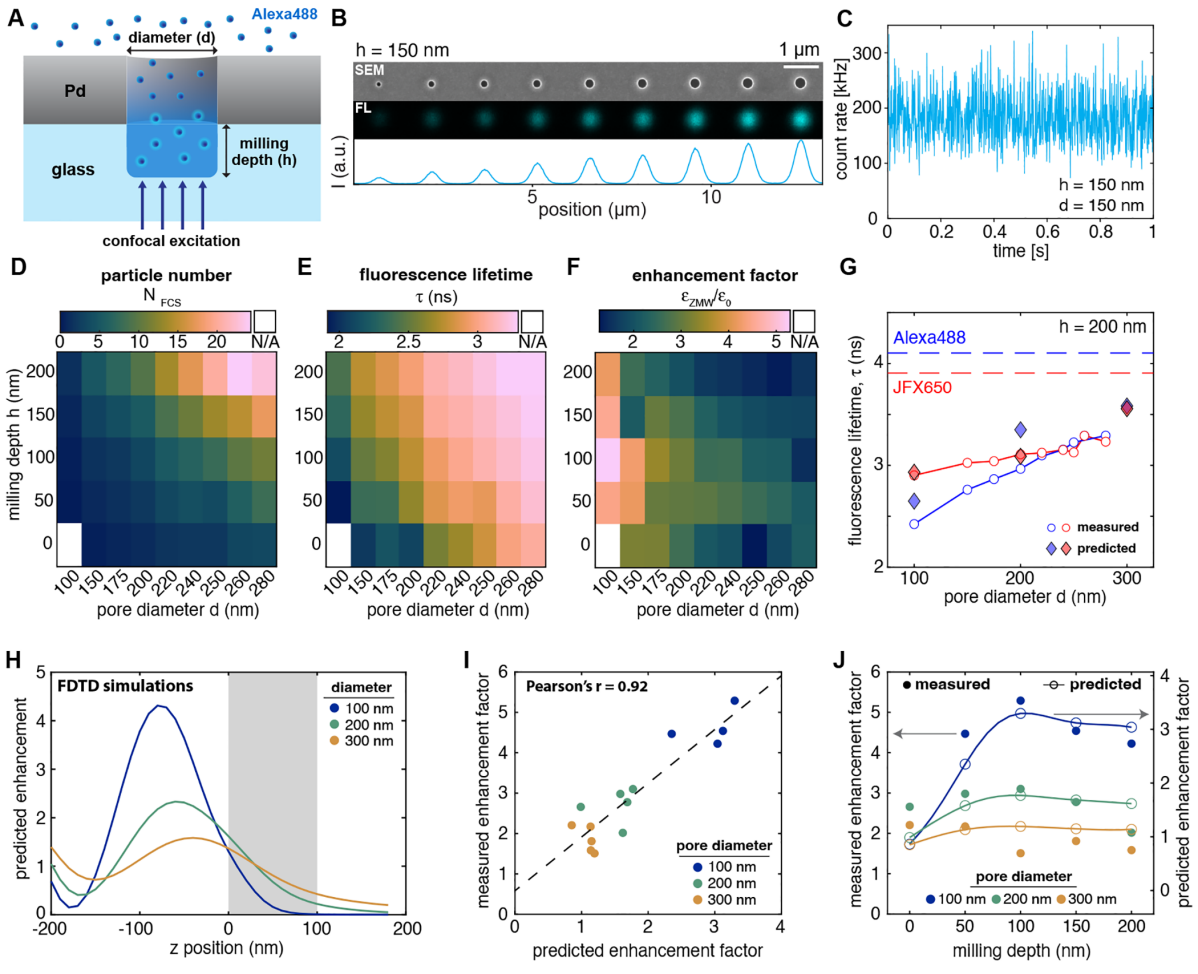
Experimental characterization of fluorescence properties in ZMWs. (A) Schematic of a ZMW with freely diffusing Alexa488 dye. (B) SEM (top) and confocal fluorescence (middle) images of a pore array with a milling depth of 150 nm. The fluorescence image was acquired at a 1 μM concentration of Alexa488. The intensity profile of the fluorescence image is shown below. The scale bar corresponds to 1 μm. (C) Example fluorescence time trace (binning: 1 ms) acquired at a concentration of 500 nM Alexa488 for a ZMW with a diameter of 280 nm and no overmilling (*h* = 0 nm). (D–F) Heatmaps of the average number of particles in the observation volume *N*_FCS_, fluorescence lifetime τ, and signal enhancement factor defined as the ratio of the counts per molecule in the ZMW compared to free diffusion, ε_ZMW_/ε_0_, acquired for 500 nM of Alexa488. Data marked as N/A could not be quantified due to insufficient signal. (G) Comparison of measured and predicted fluorescence lifetimes from FDTD simulations for an overmilling depth of 200 nm. The lifetimes of the free dyes are shown as dashed lines. (H) Predicted signal enhancement compared to a free-diffusion experiment as a function of the *z* position obtained from FDTD simulations (see Supplementary Figure 19 for details). (I) Linear regression of the measured *versus* the predicted signal enhancement. (J) Comparison of measured and predicted enhancement factors as a function of the overmilling depth.

To corroborate the theoretical results, we performed measurements on freely diffusing dyes in water in the nanowells using confocal excitation; see Figure 3A. As expected, the detected fluorescence signal of the dyes Alexa488 and JFX650 in the nanowells increased with both the pore diameter and milling depth (Figure 3B, Supplementary Figure 13 and Figure 3C,H,M, Supplementary Figures 14, 15). Time traces of the signal within the wells showed fluctuations originating from the diffusion of fluorophores (Figure 3C). Using fluorescence correlation spectroscopy (FCS), we estimated the number of particles within the nanowells, which ranged between 0 and 20 (Figure 3D, Supplementary Figures 14, 15). We observed a linear scaling of the particle number with the milling depth and quadratic scaling with the pore diameter as expected for the cylindrical wells (Supplementary Figure 16A,B). While the corresponding volumes scaled well with predictions, absolute volumes estimated by FCS exceeded the volume of the nanowells including the ZMW volume by a factor of 2–6 (Supplementary Figure 16C,D). Comparable deviations had also been observed in previous studies^7,8,12,38^ and were attributed to the signal contribution of many dim fluorophores from the highly concentrated solution that leaks through the ZMW from the other side.^7^ This explanation is further supported by the fact that the volume mismatch is largest at high volumes (Supplementary Figure 16 C,D), since large ZMW diameters showed a higher background signal also in FDTD simulations (Figure 2G–I, Supplementary Figure 12). The residence time of the dyes within the nanowell, as seen from the decay of the FCS curves, likewise increased with the size of the well (Supplementary Figures 13 and 14).

To gauge the amount of radiative and nonradiative rate enhancement experienced by the dyes, we quantified the excited state fluorescence lifetime (Figure 3E), where a reduced lifetime indicates a stronger enhancement of either radiative or nonradiative relaxation. Fluorescence decays were well described by a monoexponential model function (Supplementary Figure 13). We observed the strongest modulation of the fluorescence lifetime for small pore diameters and shallow wells where the dye is restricted within the proximity of the metal aperture. The predicted signal-averaged fluorescence lifetimes from the FDTD simulations showed excellent quantitative agreement with the experimental values (Figure 3G).

To test for a potential enhancement of the signal emanating from the nanowells, we define a signal enhancement factor by comparing the molecular brightness of the fluorophore (as measured from FCS analysis) within the nanowell to the free-diffusion value. For both dyes, a signal enhancement of 2–5 was observed across the entire parameter space (Figure 3F, Supplementary Figure 17). The largest enhancement was observed at small pore diameters, reaching a maximum value of 4–5 at a pore diameter of 100 nm and a milling depth of 100 nm for Alexa488. Higher molecular brightness correlated with a reduced fluorescence lifetime (Supplementary Figure 18). Notably, the highest signal enhancement was not obtained at zero overmilling, where the dyes are confined to the ZMW, but rather increased as the nanowell extended into the glass up to a depth of 100 nm, after which the signal enhancement was reduced.

To understand the observed enhancement, we computed the theoretical enhancement factor from the FDTD simulations (Figure 3H, Supplementary Figure 19). This enhancement originates predominantly from a focusing of the excitation light due to the formation of the standing wave, as no quantum yield enhancement was found to be present due to losses to the metal nanostructure (Supplementary Figure 10). Accordingly, the *z*-profile of the enhancement factor is dominated by the profile of the excitation field. Experimental and predicted enhancement factors showed excellent correlation, although the experimental enhancement factors slightly exceeded the predicted values (Figure 3I). The predicted enhancement factors well reproduced the experimental trends obtained for different pore diameters and milling depths, confirming the maximum of the enhancement at a depth of approximately 100 nm (Figure 3J).

In summary, overmilled palladium ZMWs provide an excellent confinement of the detected signal to the volume in the nanowell below the metal aperture for pore diameters up to 300 nm, while simultaneously offering up to a 5-fold signal enhancement by focusing the excitation power within the nanowell.

### Membrane Protrusions of Live Cells Can Be Imaged through Pd ZMWs

**Figure 4:**
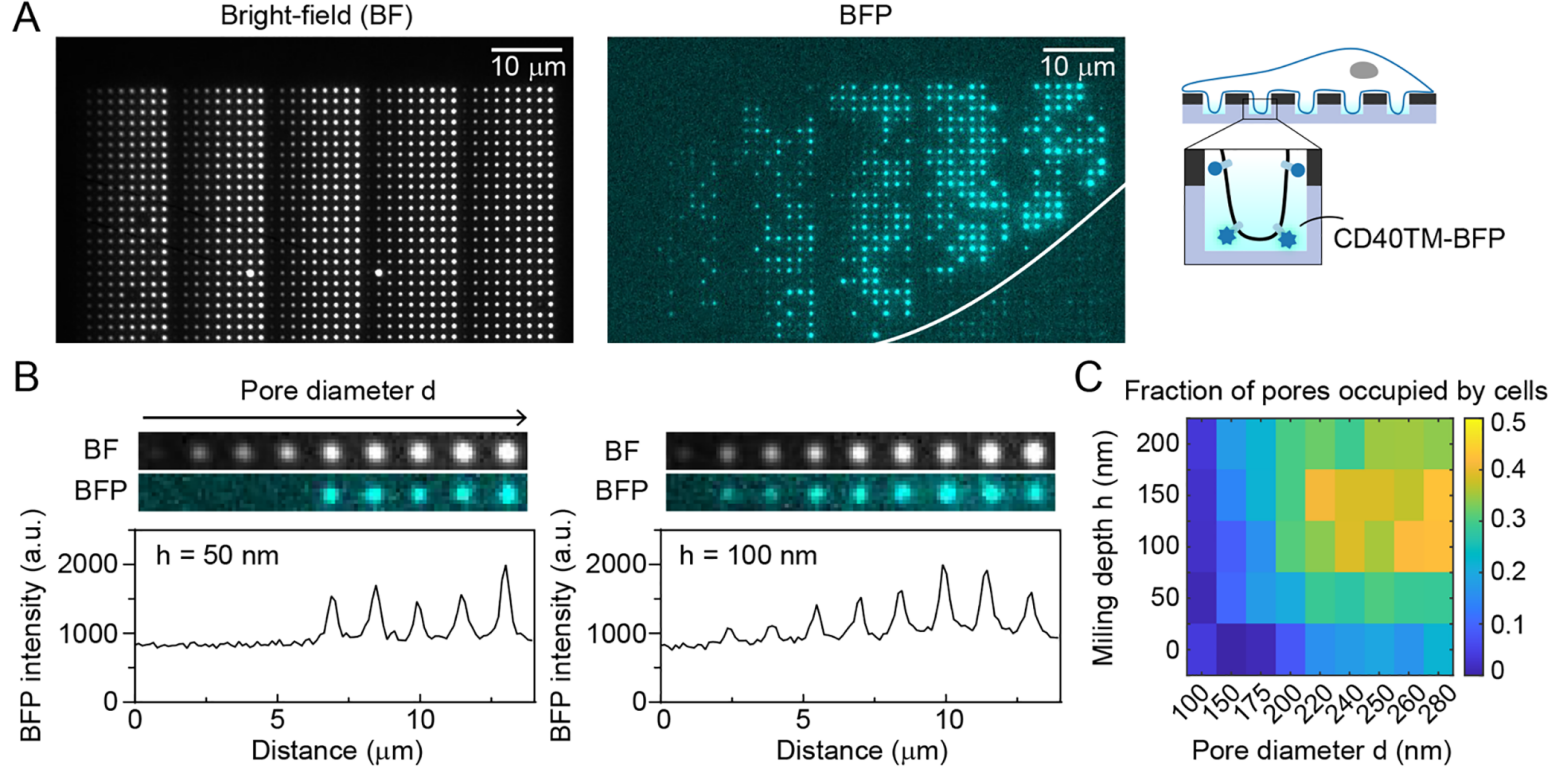
The cell membrane protrudes into nanowells. (A) U2OS cells expressing CD40TM-BFP on a ZMW array of version 1. Bright-field (left) and BFP fluorescence (right) images are shown. White outline represents an estimated outline of a cell on the surface. (B) Intensity profiles of BFP fluorescence along different pore diameters (100 nm to 280 nm) for a milling depth of 50 nm (left) and 100 nm (right). (C) Fraction of pores with detectable BFP signal for each pore size, as determined from 7367 pores potentially covered by cells.

To investigate the extent of cell membrane protrusion into ZMWs, we fluorescently labeled cell membranes using a blue fluorescent protein (BFP) fused to the transmembrane domain of the transmembrane protein CD40 (CD40TM-BFP). Cells expressing CD40TM-BFP were grown on a Pd film containing arrays of ZMWs with different diameters and depths (Supplementary Figure 1). Based on their morphology, cells appeared healthy on Pd surfaces for at least 2 days. When imaged through the ZMWs, we observed a BFP signal in many nanowells (Figure 4A). To verify the presence of cells on the ZMW arrays, coverslips were flipped upsidedown to image the entire cells using wide-field fluorescence microscopy (Supplementary Figure 22). The location of BFP-positive nanopores corresponded well to the position of cells on the nanopore array, indicating that the observed fluorescence in nanopores originated from cells that had membrane protrusions in the pores. To examine the relationship between pore size and cell membrane protrusion into pores, the BFP fluorescence intensities inside nanowells were analyzed for different pore sizes (Figure 4B, Supplementary Figure 22C). The amount of protrusions into nanowells, as assessed from the BFP signal, decreased with both decreasing pore diameter and milling depth, with the smallest pore diameter (*d* = 100 nm) only showing a very small amount (few percent) of occupied pores (Figure 4C). Efficient cell protrusions into nanowells with an occupancy of up to 50% occurred for pore diameters above 200 nm and milling depths above 50 nm. Importantly, the low occupancy for pores with no overmilling confirmed that the creation of nanowells with overmilling is key to the efficiency of the cell protrusions. Increased area of the glass surface in overmilled nanowells may facilitate the cell adhesion within the nanowells, leading to improved protrusion into nanowells compared to ZMWs without overmilling

**Figure 5:**
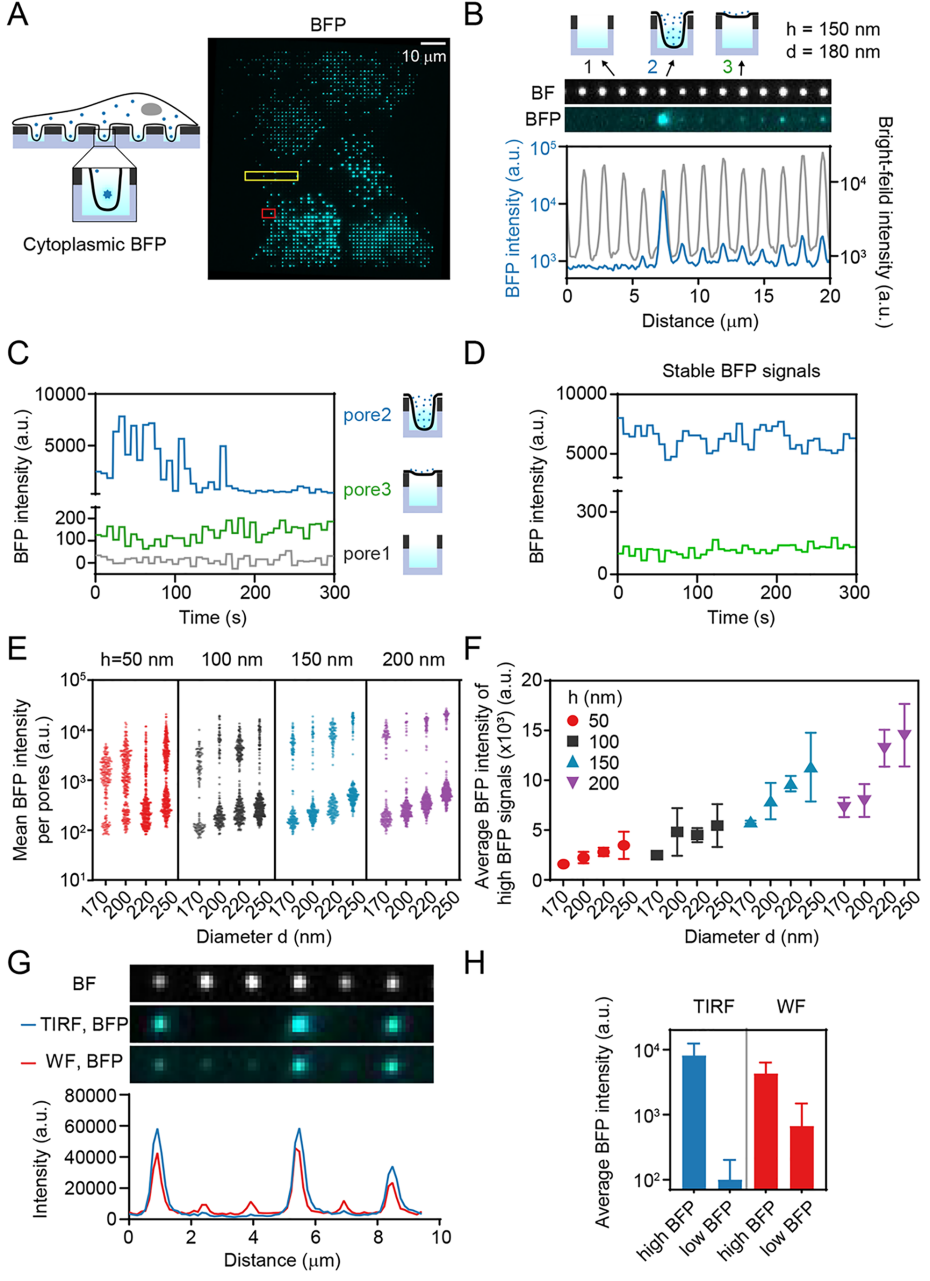
Imaging of single fluorophores in live cells that protrude into nanowells. (A) U2OS cells expressing cytoplasmic BFP were grown on the ZMW arrays of version 2 and imaged using transmission light (left) or BFP fluorescence (right). Scale bar: 10 μm. (B) Intensity profiles of BFP and transmission light for pores in the yellow box in (A). (C) BFP fluorescence time traces of the pores are indicated in B. (D) Representative time traces of BFP intensity for pores with stable high (blue line) or stable low (green line) BFP signal intensity. The pores correspond to the pores denoted by the red box in A. Images were acquired every 7.5 s. (E) Each dot represents the average BFP intensity of a time trace for an individual pore showing a stable signal. There are two distinct populations for each pore size. The number of measurements per pore size ranges from 113 to 413. (F) Average BFP intensity (mean ± SD from 3 independent experiments) of the high BFP signals in E. (G) BFP intensity profiles under wide-field and TIRF illumination. Under TIRF illumination, peak intensities are increased, while background levels are reduced. (H) Average BFP intensities of high- and low-intensity pores under TIRF or WF illumination. Error bars represent the standard deviation.

Having established that cells protrude into the nanowells, we assessed the stability and dynamics of the signals from the cell protrusions. For this, we generated a monoclonal cell line that expressed cytoplasmic BFP to assess whether cytoplasmic proteins could be imaged on experimentally relevant time scales within the nanowells (Figure 5A). Based on the efficiency of cell protrusion into nanopores of different sizes (Figure 4C), we narrowed the range of pore diameters down to 170 nm to 250 nm (Supplementary Figure 1). We observed BFP cytoplasmic fluorescence of varying intensity in a large proportion of the pores (Figure 5A, Supplementary Figure 23). To assess the stability of the cell protrusions into nanowells, we monitored the BFP signal within individual pores over time (Figure 5C,D). For approximately 50% of pores with BFP signal, the intensity remained largely constant over the acquisition time of 5 minutes, during which no significant photobleaching of the BFP signal occurred (Supplementary Figure 21). However, for a subset of pores, large fluctuations of the BFP intensity were observed, suggesting movement of cell protrusions in and out of the observation volume (Supplementary Figure 24). The occurrence of pores with switching signal did not depend on the pore diameter (Supplementary Figure 24D,E). When analyzing BFP intensities among different pores, we found a clear bimodal distribution for all pore sizes, with BFP-positive pores either having a high or low BFP signal (with the latter showing <10% of the signal obtained for pores with high intensity, Figure 5B,E). Shallow pores (*h* = 50 nm) exhibited such a stable high-intensity signal more frequently compared to deeper pores (Supplementary Figure 24C), suggesting that the pore depth influenced the stability of cell protrusions into the nanowell. We hypothesized that the high BFP intensities originated from pores containing well-defined cell protrusions, while low intensities reflected pores that were covered by a cell but in which cells did not insert a protrusion (see cartoons in Figure 5B). The signal from pores with low BFP intensities then would reflect the fluorescence signal originating from above the ZMW. In our simulations, we found that oblique angle illumination (*i*.*e*., TIRF) reduced light penetration through the ZMWs and could thus, in theory, reduce this cytoplasmic signal leaking through the ZMWs (Figure 2F–I, Supplementary Figure 12). To test this, we measured BFP intensities under both wide-field and TIRF illumination. The BFP intensity for pores with low signal was indeed markedly reduced when pores were imaged under TIRF illumination compared to wide-field illumination (Figure 5H, Supplementary Figure 25). The average BFP intensity of pores showing a stable high-intensity BFP signal positively correlated with pore size (Figure 5F), again indicating that the BFP intensity represents the cytoplasmic volume inside the pore. The results support the hypothesis that pores with low BFP signal were covered by cells, but their membrane did not penetrate into the pores, while the nanowell was occupied by a cell protrusion in the case of a high BFP signal.

### Cytoplasmic Background Can Be Efficiently Suppressed Using Pd ZMWs

**Figure 6:**
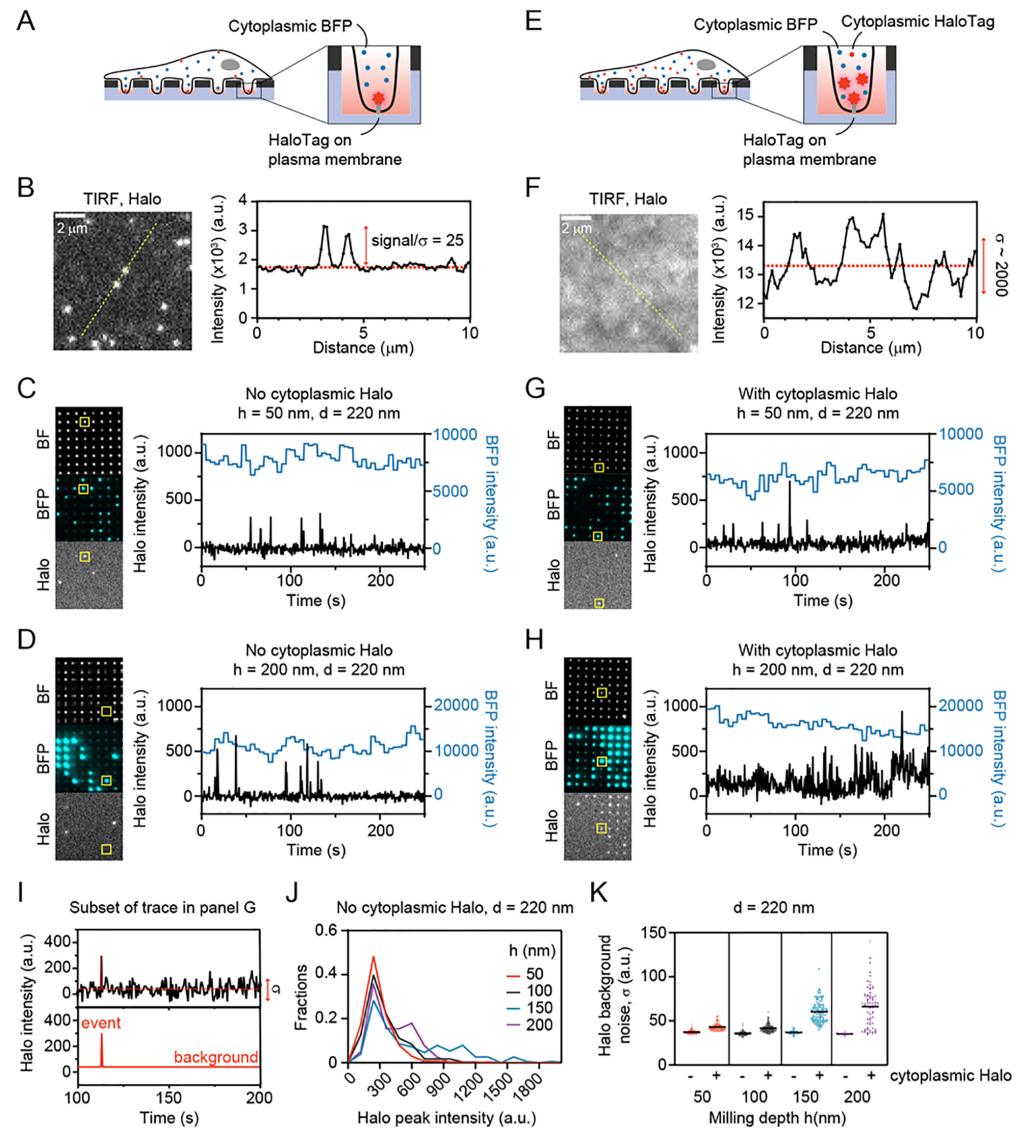
Suppression of cytoplasmic background signal using Pd ZMWs. (A) Schematic of U2OS cells expressing cytoplasmic BFP and HaloTag-TfR localized in the plasma membrane. (B) Representative TIRF image of JFX650-labeled HaloTag-TfR. Graph on the right represents an intensity profile along the yellow line. (C, D) Representative images of bright-field (BF), BFP, and JFX650-HaloTag acquired through nanopores. Fluorescence time trace of a single pore, representing the yellow boxed area in the images. Pore diameter *d* = 220 nm; milling depth *d* = 50 nm (C) and *d* = 200 nm (D). Time interval, 5 s for BFP, 500 ms for JFX650-Halo. (E) Expression of cytoplasmic HaloTag in the same cell line as in A. (F) Representative TIRF image of JFX650-labeled HaloTag-TfR. Graph on the right represents an intensity profile through the yellow line. Scale bar: 2 μm. (G, H) Representative images of BF, BFP, and JFX650-HaloTag acquired under the same imaging condition as C and D, but from the cell line additionally expressing cytoplasmic HaloTag. Fluorescence time trace of a single pore representing the yellow boxed area in the images. Pore diameter *d* = 220 nm; milling depth *h* = 50 nm (G) and *h* = 200 nm (H). Time interval, 5 s for BFP, 500 ms for JFX650-Halo. (I) Analysis of the Halo intensity time trace using a hidden Markov model to determine the Halo peak intensity and background noise of each trace. The background noise is defined as the standard deviation (σ) of the background intensity. (J) Distribution of Halo peak intensities for pores with different milling depths and a constant pore diameter of 220 nm, obtained from the cell line without cytoplasmic HaloTag described in A. (K) Background noise σ in the red HaloTag channel from individual pores. Each dotted line represents a single pore. The mean is indicated by black bars. Number of pores: *n* = 86, 133, 116, 177, 72, 113, 14, 69 (in the same order as the graph).

Having confirmed that cell protrusions remained stable within nanopores over a time scale of minutes, we next tested whether single protein molecules could be visualized within the ZMWs. As a model, we used the transferrin receptor (TfR), a transmembrane protein involved in the delivery of iron into the cells. To achieve specific labeling of TfR, we fused it to the HaloTag, a small protein tag that can be covalently labeled with fluorescent dyes.^39^ We generated a cell line stably expressing HaloTag-TfR as well as cytoplasmic BFP. BFP was used as a marker to identify which pores were occupied by cell protrusions (Figure 6A). For single-molecule imaging, we chose the JFX650 dye as the fluorophore for labeling HaloTag-TfR (JFX650-HaloTag ligand) due to its high brightness and photostability.^40^ Conventional TIRF microscopy on glass coverslips confirmed the correct localization of the HaloTag-TfR in the plasma membrane and identified the optimal dye concentration to visualize single-HaloTag-TfR molecules (Figure 6B). Cells were then cultured on ZMWs and imaged by using TIRF illumination. HaloTag-TfR signal was monitored from pores with stable cell protrusion as evidenced by constant high cytoplasmic BFP signal over the duration of the experiment (Figure 6C,D). Only pores with high BFP signal intensities exhibited a HaloTag-TfR signal (Supplementary Figure 26), further supporting that pores with low BFP signal originated from cells lying on top of the pore without protruding into the pore.

Next, we probed whether the reduction of the observation volume provided by the ZMWs would allow the detection of single membrane-bound molecules in the presence of a high cytoplasmic background in the same detection channel. To this end, we generated a cell line that expresses high levels of freely diffusing cytoplasmic HaloTag in addition to membrane-localized HaloTag-TfR (Figure 6E). TIRF microscopy is the gold standard for reduction of fluorescence background from high levels of cytoplasmic proteins. However, at high cytoplasmic Halo expression, TIRF microscopy no longer yielded sufficient background fluorescence reduction to observe single HaloTag-TfR proteins on the membrane, as can be seen in Figure 6F. In contrast, when cells were cultured on ZMWs with a 50 nm milling depth, single HaloTag-TfR molecules could readily be observed with a signal-to-noise comparable to when no cytoplasmic HaloTag was present (Figure 6C,G). This shows that the ZMWs effectively suppressed the background signal from the cytoplasmic HaloTag and allowed observation of membrane-localized HaloTag-TfR molecules. In contrast, ZMWs with a 200 nm milling depth, which yield larger optical volumes due to increased nanowell sizes, resulted in significantly higher HaloTag fluorescence background, rendering it impossible to distinguish single HaloTag-TfR molecules from the cytoplasmic background signal (Figure 3H).

To quantify the signal-to-noise ratio of single HaloTag-TfR proteins, we applied a hidden Markov model (HMM) analysis to detect the signal spikes representing single HaloTag-TfR molecules diffusing in and out of the nanowells (Figure 6I). In the cell line without cytoplasmic HaloTag, no significant differences in the Halo peak intensity and background noise were observed across different milling depths (Figure 6J,K). However, in the cell line expressing high cytoplasmic HaloTag, the background signal significantly increased for deeper pores due to the larger cytoplasmic volume present within the observation volume (Figure 3K). These results show that the milling depth plays a crucial role for background suppression. Taken together, this work reveals that imaging cells on overmilled ZMWs allows for the visualization of single fluorescent molecules even in the presence of high fluorescent background in the cytoplasm.

## Discussion

Here, we have introduced overmilled palladium ZMWs combined with TIRF illumination as a platform for single-molecule studies in live cells. By creating an attoliter-volume size-tunable nanowell below the ZMW, we achieved a highly confined observation volume that is efficiently penetrated by cell protrusions. The resulting reduction of the cytoplasmic volume combined with favorable optical properties of the nanowell enabled the observation of single fluorescently labeled cellular membrane proteins even in the presence of high cytoplasmic concentrations of the same fluorophore.

We demonstrated an effective confinement of the observation volume to the nanowell, a strong rejection of background fluorescence originating from the other side of the ZMW, and an up to 5-fold signal enhancement, both theoretically using FDTD simulations and by *in vitro* experiments of freely diffusing fluorophores (Figure 2 and Figure 3). Significant nonradiative losses to the metal occurred for shallow milling depths and small pore diameters where the dyes are confined to the proximity of the metal, as evidenced by a strong decrease of the excited state lifetime of the fluorophore, reaching up to a 2.3-fold reduction. Similar changes of the fluorescence lifetime were reported for aluminum^8,37^ and aluminum/gold alloy ZMWs,^13^ with a 2- to 6-fold reduction of the fluorescence lifetime for various fluorophores and ZMW diameters. Due to a lack of radiative enhancement, this results in a strong quantum yield reduction of up to 4-fold within ZMWs with small pore diameters, which however approaches the free dye value quickly with increasing distance from the metal (Figure 2B).

Despite an ≈10% reduction of the quantum yield throughout the observation volume, the signal from within the nanowells is enhanced by a factor of 2 to 5 due to the combination of two effects. First, the excitation intensity is focused within the nanowell due to the formation of a standing wave below the metal layer that leads to a 3-fold increase of the excitation intensity at its maximum (Figure 2 F^36,37^). Second, an approximate 2-fold increase of the detection efficiency arises because the emission is guided toward the detection side due to the reflective metal surface (Figure 2E). The conclusions reached here for the green detection channel using the dye Alexa488 (λ_ex_ = 488 nm, λ_em_ = 525 nm) apply also to the far-red detection channel using the dye JFX650 (λ_ex_ = 640 nm, λ_em_ = 670 nm), from both the theoretical (Supplementary Figures 8, 9) and experimental side (Supplementary Figure 17), and are thus expected to remain valid over the whole visible spectrum. While similar results are expected also for other metals such as gold and aluminum,^8−10,12,13,35−38^ palladium offers a simpler fabrication process compared to aluminum by eliminating the need for a SiO_2_ passivation layer and a reduced photoluminescence in the green spectral range compared to gold,^23^ which overlaps with the emission of the widely used GFP tag.

Our insights into the optical properties of the nanowells have a number of consequences for *in vitro* and *in cellulo* applications of ZMWs. For standard ZMWs without overmilling, the zeptoliter-size observation volume results in very short dwell times of freely diffusing molecules in the observation volume, necessitating immobilization of the molecules of interest. Using overmilled ZMWs, we achieve dwell times for freely diffusing molecules that are comparable to residence times in a diffraction-limited confocal volume, enabling potential applications of overmilled ZMWs in single-molecule spectroscopy and single-molecule FRET experiments.^41,42^ The larger volume of the overmilled ZMWs also provides the possibility to study large and flexible molecules, such as extended DNA/RNA molecules, that are otherwise difficult to confine to a small volume. Furthermore, the distance-dependent quenching by the metal can lead to a significant signal reduction in standard ZMWs,^33^ which is avoided in overmilled ZMWs where molecules are at sufficient distance from the metal. The excitation intensity within standard ZMWs also generally remains limited when molecules are not directly immobilized on the glass surface due to the exponentially decaying evanescent field. This situation is resolved in overmilled ZMWs where the excitation power is effectively focused to the nanowell due to the formation of a standing wave, an effect that cannot be exploited in standard ZMWs. While an increase of the excitation intensity can also be achieved by increasing laser powers, the distinctive intensity distribution within the nanowell further improves the background suppression by preferential excitation of molecules below the ZMW. Lastly, the metal layer acts as a mirror surface that leads to a more efficient collection of the fluorescence emission from within the nanowell. This provides a more efficient use of the limited signal in single-molecule experiments, which is especially crucial in live cell applications, where photostabilization by oxygen scavenging and use of reducing–oxidizing agents is difficult. A drawback of nanowells compared to standard ZMWs is that a larger observation volume in nanowells limits single-molecule detection sensitivity when very high protein concentrations are used (micromolar–millimolar). Therefore, standard ZMWs and nanowells will each have their specific applications, with nanowells performing especially well in cell-based imaging.

Pd-based ZMW nanowells showed excellent compatibility with live-cell imaging. Cells grew readily on palladium-coated glass coverslips, adhered to the untreated metal surface, and showed healthy morphologies. Efficient protrusion into the nanowells was observed for most pore sizes, except for the smallest diameter of 100 nm, with the frequency of membrane protrusions increasing both with milling depth and pore diameter, in agreement with previous results on Al ZMWs.^31^ Time-dependent fluctuations of the fluorescence signals of cytoplasmic BFP confirmed that cells dynamically explored the nanowells over a time scale of minutes (Figure 5C,D). Approximately 10% of pores exhibited significant cytoplasmic signal that remained stable over a time scale of 5 min, showing that cells formed stable protrusions into the nanowells on relevant time scales for many biological processes. While the cytoplasmic background signal increased with pore size (Figure 5E, in agreement with ref (31)), it was markedly reduced under TIRF illumination due to a reduced propagation of the excitation light through the ZMW and a more even excitation intensity within the nanowell (Figure 5H, Figure 2F–I, Supplementary Figure 12).

We showed the applicability of the nanowells for single-molecule fluorescence experiments in live cells and their superiority compared to TIRF microscopy with respect to single-molecule observations. By following the fluorescence of single membrane-bound fluorophores, we found that signal spikes originating from single molecules were only observed for pores with stable protrusions. Most importantly, single-molecule signals could still be observed under conditions of high cytoplasmic expression, which did not allow for single-molecule experiments using conventional TIRF excitation (Figure 6F–H). Despite the high concentration of fluorophores in the cytoplasm, we could achieve a signal-to-noise ratio of ≈7 for shallow pores up to a depth of 100 nm, with noise levels equivalent to when no cytoplasmic signal was present (Figure 6J,K). The results confirm the excellent suppression of the cytoplasmic signal provided by the ZMW nanowells, allowing monitoring of single fluorophores despite a high cytoplasmic background of the same fluorophore.

We envision that overmilled ZMWs will allow live-cell single-molecule fluorescence experiments in a wider range of cases. More specifically, it provides a benefit in cases where expression levels cannot be controlled, for example, when studying endogenously expressed proteins of interest or when weak interactions are studied requiring high concentrations of the interaction partners. While we have only tested the human osteosarcoma U2OS cell line in this study, live-cell ZMW imaging has been applied successfully in other cell lines, including COS-7 cells,^30^ rat basophilic leukemia mast cells,^31^ and mouse neuroblastoma N2a cells,^32^ suggesting its potential for broad applications. To facilitate cell protrusion into the nanopores, surface coatings or functionalization with specific molecules or peptides can be employed as effective strategies,^29,43,44^ which may extend the application further to a variety of cell types. Since the total cellular membrane fraction that protrudes into the nanowells is small (≤1%), efficient approaches for membrane recruitment and immobilization of low-concentration complexes will be required. A potential strategy could be through introduction of a designed transmembrane protein with an intracellular docking platform and extracellular binding to the glass wells below the Pd layer using silane chemistry^45^ or electrostatic interactions with the glass surface using polylysine.^44,46^ Alternatively, higher throughput could be achieved by employing fabrication methods based on electron-beam lithography^47^ with subsequent wet etching.^48^ We envision many applications of our method for the study of protein–protein and protein–RNA interactions in the cytoplasm, enzymatic activities of single proteins or complexes such as ribosomes^49^ or proteasomes,^50^ and protein conformational dynamics by combination with single-molecule FRET.^41^ In this study, we focused on membrane-associated proteins, which have a longer residence time within the nanowells compared to that of cytoplasmic fluorophores. Nonetheless, it is likely that ZMW nanowells can similarly be adopted to study freely diffusing molecules, which would increase the applicability of overmilled ZMWs even further.

## Conclusions

We introduced the use of overmilled zero-mode waveguides made of palladium combined with TIRF illumination for live-cell imaging. We performed a thorough theoretical and experimental characterization of the optical properties of the nanowells using FDTD simulations and fluorescence experiments of freely diffusing organic dyes, which together delineated the signal confinement and fluorescence enhancement within the overmilled nanowell volume. Live-cell experiments showed that cells readily protrude into the nanowells, enabling single-molecule fluorescence experiments with excellent signal-to-noise ratio despite a high cytosolic concentration of the fluorophore. By scanning a wide range of pore diameters and milling depths, we provided comprehensive guidelines for future *in vitro* or *in cellulo* single-molecule studies where a compromise must be found between the required background suppression, the desired fluorescence enhancement, and the efficiency of cell protrusions.

## Methods and Materials

### Fabrication of Palladium Zero-Mode Waveguides

Standard borosilicate coverslips (no. 1.5H, Marienfeld, Germany) were cleaned by consecutive sonication in deionized water, isopropyl alcohol, acetone, and 1 M potassium hydroxide solution, washed with deionized water, and spin dried. A thin adhesion layer of 3 nm titanium was deposited at a rate of 0.05 nm/s under a base pressure of 3 × 10^–6^ Torr in a Temescal FC2000 e-gun evaporator. In the same vacuum, a layer of Pd was immediately added on top of the Ti. Two versions of Pd ZMWs were used in this study and differed by the thickness of the Pd layer. Either 100 nm of Pd was deposited at a rate of 0.1 nm/s (version 1) or 150 nm at a rate of 0.2 nm/s (version 2), both under a base pressure below 2 × 10^–6^ Torr.

Nanopores were milled through the layers *via* FIB milling on a FEI Helios G4 CX FIB/SEM. To improve consistency, the focus and stigmation of the ion beam were optimized on a graphite standard sample before milling. For the pore arrays of version 1, a 33 pA beam with an acceleration voltage of 30 kV was used. For the pores of version 2, the beam current was set to 430 pA at the same voltage. Due to the higher beam current used for version 2, milling time was reduced to around 4 min per array coming at the cost of less well-defined pore diameters (Supplementary Figure 1). The diameters of the resulting pores were measured using the immersion mode of the scanning electron microscope on the same machine. The depth and opening angle of the pores, resulting from overmilling into the glass surface, were measured by cutting through the pores with the ion beam and imaging under a 52° incident angle. The diameters, depths, and taper angles of the pores can be found in Figure 1C,D and Supplementary Figure 1C–F.

Prior to experiments, ZMWs were thoroughly cleaned by consecutive sonication in deionized water, ethanol, isopropyl alcohol, acetone, and 1 M potassium hydroxide solution for about 10 min each and exposed to oxygen plasma at a power of 90 W for 1 min. The coverslips could be reused about 10 times, after which the Pd film started to show signs of degradation.

### Single-Molecule Measurements of Free Fluorophores

Measurements of freely diffusing fluorophores inside Pd ZMWs were performed on coverslips of version 1 on a Picoquant Microtime 200 microscope operated using the Symphotime software in a temperature-controlled room at 21.5 ± 1.0 °C. Lasers were focused by an 60× Olympus UPSLAPO 60XW water immersion objective with a working distance of 280 μm and a numerical aperture of 1.2. Excitation at wavelengths of 640 nm and 485 nm was performed at powers of 10 μW as measured at the sample plane. Pulsed lasers were operated in pulsed interleaved excitation at a repetition frequency of 40 MHz.^51^ The molecular brightness of a solution of Alexa488 fluorophores was optimized by adjusting the correction collar of the objective prior to the experiment. The emission light was passed through a 50 μm pinhole, split by a dichroic mirror, and was filtered by 525/50 or 600/75 optical band-pass filters for the blue and red detection channels, respectively (Chroma, Bellow Falls). Fluorescence emission was detected on single-photon avalanche-diode detectors (PD5CTC and PD1CTC, Micro Photon Devices, Bolzano). Fluorophore solutions of 500 nM of Alexa488 and JFX650-HaloTag were supplemented by 0.1% Tween20 (Fisher Scientific) to minimize surface adhesion of the fluorophores. Transmission light images of the pore arrays were used to locate the pores prior to fine-tuning the position of the laser focus to maximize the signal.

### FDTD Simulations of Light Fields inside Pd ZMWs

Three-dimensional FDTD simulations were performed using Lumerical FDTD (ANSYS Inc., USA) as described previously for the characterization of free-standing ZMWs^24^ and reiterated here for convenience of the reader. The surrounding medium was modeled as water with a refractive index of 1.33, and the refractive indices of the 100 nm thick palladium membrane and the SiO_2_ layer were modeled according to ref (52). For the simulation of the excitation field, the ZMW was illuminated by a total-field scattered-field source that was polarized in the *x*-direction. The source was set as a plane wave for wide-field excitation and TIRF excitation under an incidence angle of 70° and a Gaussian source with a numerical aperture of 1.2 for focused excitation. The simulation box size was 1 × 1 × 0.8 μm^3^ for wide-field and TIRF excitation with a grid resolution of 5 nm. To correctly model the focused beam, a larger box of 4 × 4 × 0.8 μm^3^ was required for the Gaussian source, in which case a larger grid resolution of 50 nm was used to model the field further away from the nanowell, keeping the 5 nm grid resolution close to the nanowell. The electromagnetic field intensity distributions, computed as the absolute value of the complex electric field, |*E*|^2^, in the *xz*- and *yz*-planes at the center of the nanowell and in the *xy*-plane at the ZMW entry, are shown in Figure 2 and Supplementary Figures 2–7. To model the fluorescence emission, a dipole emitter was placed at varying *z*-positions along the central axis of the nanowell. The radiated power was monitored on all sides of the simulation box (see below). To compute the detection efficiency, the radiated power was integrated only on the detection side below the palladium layer. For the dipole emission, all reported quantities were averaged over horizontal and vertical orientations of the dipole to model isotropic emission. The power was only weakly affected by the lateral position of the emitter with respect to the center of the nanowell (Supplementary Figure 11^7^).

#### Estimation of Quantum Yield and Fluorescence Lifetimes

Quantum yields and fluorescence were computed as described previously.^24^ For the convenience of the reader, we repeat this description here. In the absence of the nanostructure, the decay rate of the excited molecule is given by γ^0^ = γ_r_^0^ + γ_nr_^0^, where γ_r_^0^ and γ_nr_^0^ are the radiative and nonradiative decay rates. Here, γ_nr_^0^ represents the rate of nonradiative relaxation to the ground state due to internal processes, which is assumed to be unaffected by the nanostructure. The intrinsic quantum yield of the fluorophore is defined as Φ_0_ = γ_r_^0^/(γ_r_^0^ + γ_nr_^0^) and was obtained from the literature as Φ_0_ = 0.8 and 0.53 for Alexa488 and JFX650.^40,53^

Within the nanowell, the radiative decay rate γ_r_ is modified. Additionally, a nonradiative loss rate γ_loss_ arises due to absorption by the metal nanostructure.^54^ The quantum yield Φ in the presence of the ZMW is given by^55^
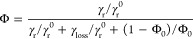
1where γ_r_^0^ and γ_r_ are the radiative rates in the absence and the presence of the ZMW, respectively. While absolute decay rates γ_r_, γ_loss_, and γ_r_^0^ are inaccessible from FDTD simulations, relative rates normalized to the radiative rate in the absence of the ZMW, γ_r_^0^, can be obtained from the power *P* radiated by the dipole^56^ as
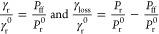
2where *P*_r_ and *P*_r_^0^ are the powers radiated by the dipole in the presence and absence of the ZMW, and *P*_ff_ is the power that is radiated into the far-field in the presence of the ZMW. See Figure 2and Supplementary Figure 10 for the obtained *z*-profiles of the normalized radiative and nonradiative rates.

To obtain the fluorescence lifetime τ, which is given by the inverse of the sum of all de-excitation rates, we use the relation τ = Φ/γ_r_ in combination with eq 1:
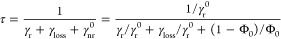
3where the intrinsic radiative rate γ_r_^0^ was estimated as γ_r_^0^ = Φ_0_/τ_0_, with the experimentally measured fluorescence lifetimes τ_0_ for Alexa488 and JFX650 of 4.0 ns and 3.9 ns. The detection efficiency η was estimated as the fraction of the power radiated toward the lower (detection) side of the ZMW, *P*_ff_^*z*–^, with respect to the total radiated power:
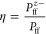
4

Finally, the total detected signal as a function of the *z*-position of the emitter within the nanowell was computed as the product of the excitation intensity *I*_ex_(*z*), detection efficiency η(*z*), and quantum yield Φ(*z*) as
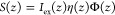
5

The computed detection efficiency η, quantum yield Φ, detected signal *S*(*z*), and lifetime τ as a function of the *z*-position within the ZMW are shown in Supplementary Figures 8, 9.

#### Estimation of Signal Enhancement Factors

To estimate the theoretical signal enhancement factor, we performed simulations in the absence of the palladium layer and glass nanowell to mimic the free diffusion experiment (Supplementary Figure 19). A 50 nm thin glass layer was added at the edge of the simulated volume (at *z* ≈ −4 μm) to account for the glass–water interface. The signal enhancement factor at each *z*-position was computed as the ratio of the detected signal in the nanowell and the free diffusion value:
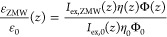
6where Φ_0_ is the reference quantum yield. Here, we assume that 50% of the signal is detected in the free diffusion case (η_0_ = 0.5) and neglect detection losses due to the limited numerical aperture of the objective lens, which are assumed to be identical for the compared conditions. The predicted average enhancement factors were then computed as the signal-weighted average along the central pore axis (*x* = 0, *y* = 0) from the bottom of the well of depth *h* toward the end of the simulation box at height 200 nm:
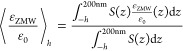
7where *S*(*z*) is the detected signal as defined in eq 5. See Supplementary Figure 19 for details.

### Live Cell Imaging on Pd ZMWs

#### Cell Lines

Human U2OS (ATCC, HTB-96) and HEK 293T (ATCC, CRL-3216) cells were used for imaging and lentivirus production, respectively. They were grown in DMEM (4.5 g/L glucose, Gibco) with 5% fetal bovine serum (Sigma-Aldrich) and 1% penicillin/streptomycin (Gibco) and maintained at 37 °C with 5% CO_2_. The cell lines were confirmed to be mycoplasma-free. Cell lines stably expressing transgenes were generated *via* lentiviral transduction. Lentivirus was produced by transfecting HEK 293T cells with polyethylenimine and packaging vectors (psPAX2, pMD2.g) and the lentiviral plasmid of interest. The viral supernatant was collected 72 h after transfection. Cells were seeded for infection at ≈35% confluency 24 h prior to lentivirus addition. The cells were spin-infected with the viral supernatant and Polybrene (10 μg/mL) for 90 min at 2000 rpm at 32 °C, then cultured for 48 h. Monoclonal cell lines expressing the transgene were isolated by single-cell sorting into 96-well plates *via* FACS. The TfR coding sequence was amplified from Addgene plasmid #133451.

#### Cell Culture for Imaging

Cells were seeded on ZMW coverslips in a six-well plate at 40% to 45% confluency 1 day before the imaging experiment. The cell culture medium was replaced with imaging medium (prewarmed CO_2_-independent Leibovitz’s-15 medium (Gibco) with 5% fetal bovine serum and 1% penicillin/streptomycin) 30 min prior to imaging. All live-cell imaging experiments were performed at 37 °C. For experiments with HaloTag-expressing cell lines, the cell culture medium was replaced with the imaging medium (prewarmed Leibovitz’s-15 medium with 5% fetal bovine serum and 1% penicillin/streptomycin) containing 5 nM JFX650-Halo ligands. After 10 min of incubation with the Halo ligands, the cells were washed three times with fresh imaging medium.

#### Microscope and Image Acquisition

Live-cell imaging experiments were performed using a Nikon TI inverted microscope equipped with a TIRF illuminator, perfect focus system, and NIS Element Software. A Nikon CFI Apochromat TIRF 100× 1.49 NA oil-immersion objective was used. The microscope was equipped with a temperature-controlled incubator. Bright-field and fluorescence images at each ZMW array position were recorded by using an Andor iXon Ultra 888 EMCCD camera.

### Data Analysis

#### Single-Molecule Fluorescence Experiments

Fluorescence correlation spectroscopy and lifetime analysis were performed using the PAM software package.^57^ Autocorrelation functions *G*(*t*_c_) were fit to a standard model function for 3D diffusion:
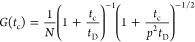
8where *t*_c_ is the correlation time, *N* is the average number of particles in the observation volume, *t*_D_ is the diffusion time, and *p* is a geometric factor that accounts for the axial elongation of the confocal volume (*p* = 3.4). While, strictly speaking, the 3D diffusion model is not applicable for the complex geometries in this study, we apply it here as a simple means to extract the amplitude and average decay time of the curves. The molecular brightness ε was calculated from the average signal ⟨*I*⟩ as ε = ⟨*I*⟩/*N*. The effective volume was computed by using the known concentration *c* of the fluorophore as *V*_FCS_ = *N*/(*N*_A_*c*), where *N*_A_ is Avogadro’s number. Fluorescence decays of Alexa488 were fitted to a single-exponential decay that was convoluted with the instrument response function. Decays for JFX650 generally required two lifetimes to achieve a good fit, of which we report the average. The second component most likely originates from a fraction of dyes that were sticking to the surface.

#### Cell Imaging

The images were analyzed using custom-written software for MATLAB. The program automatically determined the positions of pores from bright-field images and calculated the fluorescence intensity of each pore from the fluorescence images. The BFP and JFX650-Halo fluorescence signals were obtained by calculating the mean intensity over a 7 × 7 pixel area around the pore and subtracting the background intensity determined from the outer edge pixels of the pore. To analyze the fraction of pores occupied by cells for each pore size (Figure 4C), we estimated the area occupied by cells by manually determining an outline containing connected areas of occupied wells from six images. We analyzed the BFP intensity of each well in this area with a total of 7367 wells analyzed. As a negative control, we measured the BFP intensity of the pores where no cells were present and set a threshold to determine the positive BFP intensity pores. The total number of pores with a positive BFP signal above the threshold was 1890 (Supplementary Figure 22C). This panel illustrates the fraction of pores with a positive BFP intensity among the analyzed pores for each pore size. For the analysis of the JFX650-Halo time traces, a hidden Markov model (vbFRET algorithm^58^) with the default setting of the algorithm was used to assign on and off states of the Halo signal.

## Data Availability

All data underlying this study is made available in an open repository.^59^

## Supporting Information Available

Details on nanofabrication; additional visualization and analysis of FDTD simulations; exemplary fluorescence decays and FCS curves, overview of experimental observables, and further analysis of *in vitro* experiments; additional data, control experiments, and additional quantification of live-cell experiments (PDF)

## Notes

The manuscript has been previously deposited on a preprint server as Sora Yang*; Nils Klughammer*; Anders Barth*; Marvin E. Tanenbaum°; Cees Dekker°. Zero-mode waveguide nanowells for single-molecule detection in living cells. *bioRxiv*. 2023, 2023.06.26.546504.10.1101/2023.06.26.546504 (accessed September 4, 2023).
