# Supplementary material for: Zero-Mode Waveguide Nanowells for Single-Molecule Detection in Living Cells
Source: ACS Nano. 2023 Oct 4;17(20):20179–93. doi: 10.1021/acsnano.3c05959 (PMC10604100; doi:10.1021/acsnano.3c05959)
Supplement: Supplementary file 1 — nn3c05959_si_001.pdf [file nn3c05959_si_001.pdf]

Supporting Information: Zero-mode waveguide nanowells for single-molecule detection in living cells

Sora Yang [†,¶] Nils Klughammer [‡,¶] Anders Barth [‡,¶] Marvin E. Tanenbaum [*,†,‡,§] and Cees Dekker [*,‡,§]

†*Oncode Institute, Hubrecht Institute–KNAW and University Medical Center Utrecht, Uppsalalaan 8, 3584 CT, Utrecht, the Netherlands*

‡*Department of Bionanoscience, Kavli Institute of Nanoscience, Delft University of Technology, Van der Maasweg 9, 2629 HZ, Delft, The Netherlands*

¶*These authors contributed equally to this work.*

§*These senior authors contributed equally to this work.*

E-mail: m.tanenbaum@hubrecht.eu; c.dekker@tudelft.nl

- Supplementary Figure to Figure 1
  - Additional SEM images and layouts of version 1 and 2 arrays.
- Supplementary Figures to Figure 2
  - Excitation field intensity distributions from FDTD simulations under widefield excitation at $\lambda_{ex} = 488\,\text{nm}$.
  - Excitation field intensity distributions from FDTD simulations under widefield excitation at $\lambda_{ex} = 640\,\text{nm}$.

- Excitation field intensity distributions from FDTD simulations under TIRF illumination at an angle of 70° at $\lambda_{ex} = 488$ nm.
- Excitation field intensity distributions from FDTD simulations under TIRF illumination at an angle of 70° at $\lambda_{ex} = 640$ nm.
- Excitation field intensity distributions from FDTD simulations under excitation by a focused Gaussian beam at NA = 1.2 and a wavelength of $\lambda_{ex} = 488$ nm.
- Excitation field intensity distributions obtained from FDTD simulations under excitation by a focused Gaussian beam at NA = 1.2 and $\lambda_{ex} = 640$ nm.
- FDTD simulations of fluorescence emission and detected signal from overmilled ZMWs for Alexa488.
- FDTD simulations of fluorescence emission and detected signal from overmilled ZMWs for JFX650.
- Overview of radiative and non-radiative rates obtained from FDTD simulations of fluorescence emission within overmilled ZMWs.
- FDTD simulations of dipole emission as a function of the distance to the pore walls.
- Estimation of background signal from FDTD simulations for the dyes Alexa488 and JFX650 under the different excitation modes.

- Supplementary Figures to Figure 3
  - Experimental characterization of photophysics and diffusion within ZMWs.
  - Extracted parameters for the dye Alexa488 in overmilled Pd ZMWs.
  - Extracted parameters for the dye JFX650 in overmilled Pd ZMWs.
  - Quantification of the observation volume in overmilled ZMWs.
  - Comparison of extracted parameters for the dyes Alexa488 and JFX650 in ZMW.

  - Correlation between fluorescence lifetime and molecular brightness in ZMW for Alexa488 and JFX650.
  - FDTD simulations of excitation field and fluorescence emission in the absence of a Pd layer.
  - Comparison of experimental and predicted enhancement factors in overmilled Pd ZMWs.

- Supplementary Figures to Figure 4
  - Stability of the BFP signal during the experiments.
  - Correlated imaging of flipped coverslips confirms the colocalization of pores with BFP signal with the presence of cells on the ZMW array.
- Supplementary Figures to Figure 5
  - Brightfield and BFP-fluorescence images of array version 2.
  - BFP fluorescence intensity switching between high and low signal levels.
  - TIRF illumination vs. widefield illumination.
- Supplementary Figures to Figure 6
  - BFP and JFX650-HaloTag signal of pores showing low BFP signal.

Supporting Information Available

Supplementary Figure to Figure 1

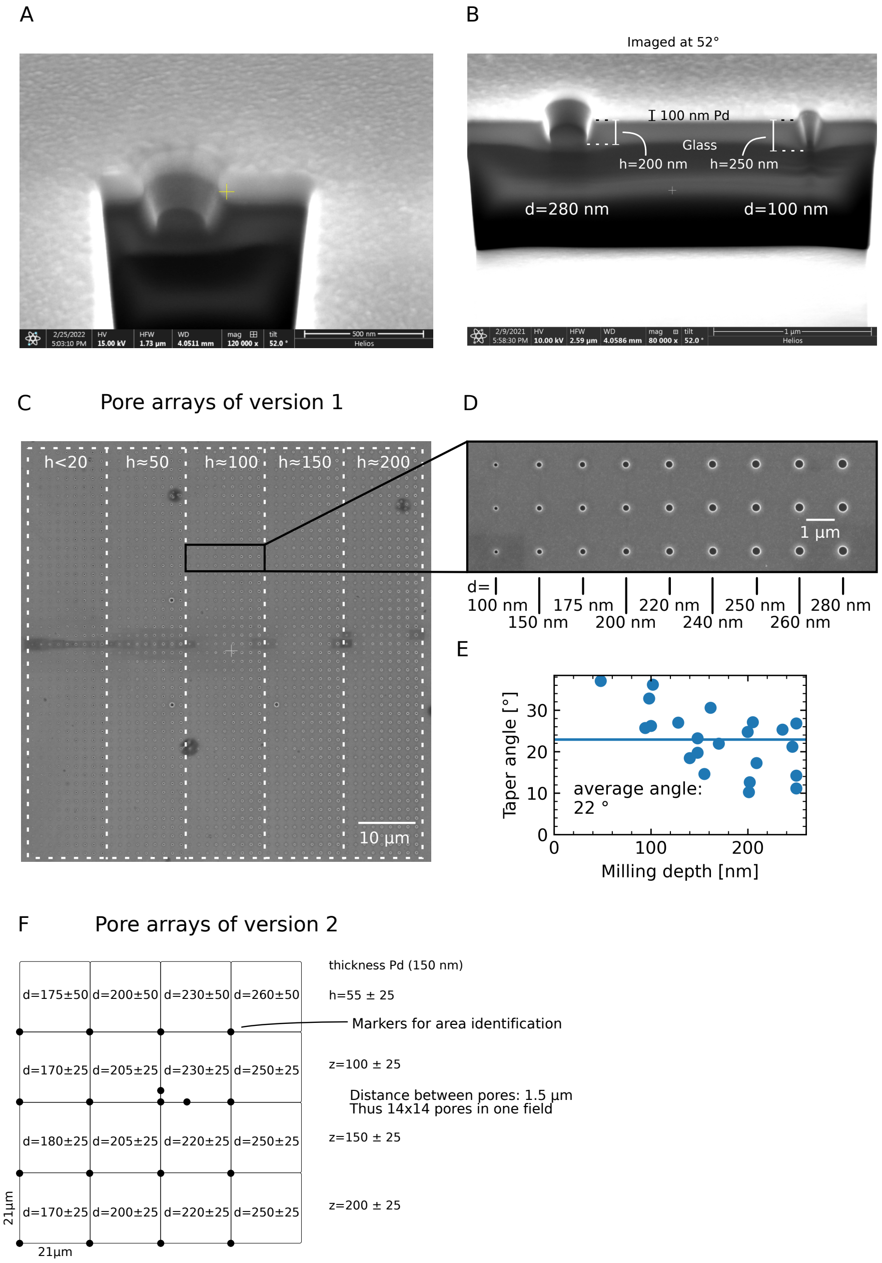

Supplementary Figure 1: **Additional SEM images and layouts of version 1 and 2 arrays. A,B:** SEM images of overmilled Pd ZMWs, imaged under 52 °. Pd shows up as a bright layer and glass as a dark layer. **C:** An SEM image of a Pd ZMW array of version 1 containing five regions of different milling depths (h), each made from nine rows of pores with varying diameter. **D:** Zoom in of C. **E:** Taper angle vs. milling depth. For deeper pores, the edges were more perpendicular with an average of 22 ° (horizontal line). **F:** Diameters (d) and depths (h) in nm of ZMW arrays for arrays of version 2 together with their uncertainty (estimated from measuring several pores).

Supplementary Figures to Figure 2

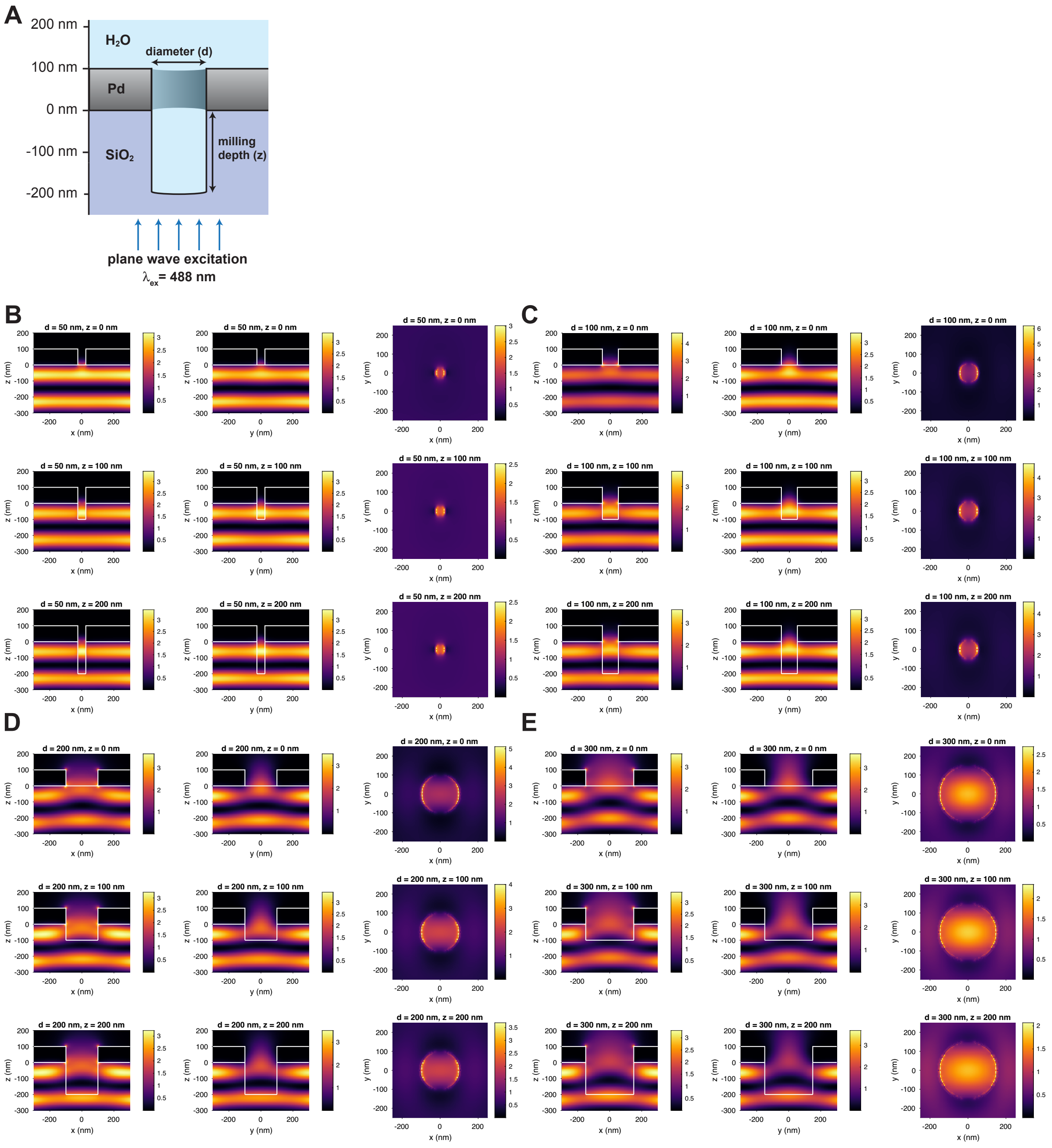

Supplementary Figure 2: **Excitation field intensity distributions from FDTD simulations under widefield excitation at $\lambda_{ex} = 488\,\text{nm}$.** **A:** Schematic of the simulation setup. **B-E:** Excitation field intensity distributions $|E|^2$ in V^2/m^2 in the x-z (left), y-z (middle) and x-y plane at the entrance to the ZMW (right) at pore diameters d of 50 nm (B), 100 nm (C), 200 nm (D), and 300 nm (E) and milling depths h of 0 nm (top), 100 nm (middle), and 200 nm (bottom). The electric field is polarized along the x-axis.

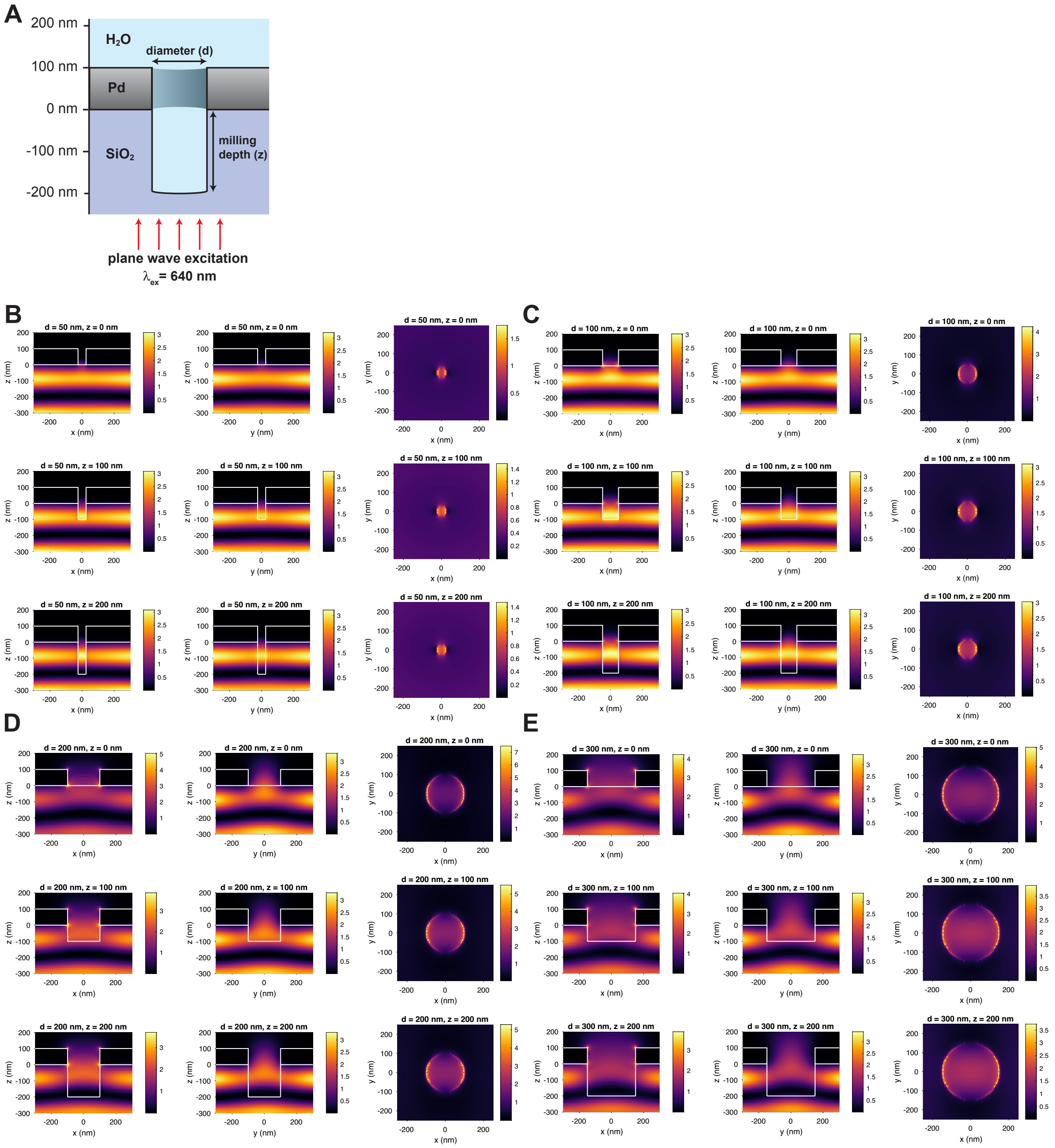

Supplementary Figure 3: **Excitation field intensity distributions from FDTD simulations under widefield excitation at $\lambda_{ex} = 640\,$nm.** **A:** Schematic of the simulation setup. **B-E:** Excitation field intensity distributions $|E|^2$ in V^2/m^2 in the x-z (left), y-z (middle) and x-y plane at the entrance to the ZMW (right) at pore diameters d of 50 nm (B), 100 nm (C), 200 nm (D), and 300 nm (E) and milling depths h of 0 nm (top), 100 nm (middle), and 200 nm (bottom). The electric field is polarized along the x-axis.

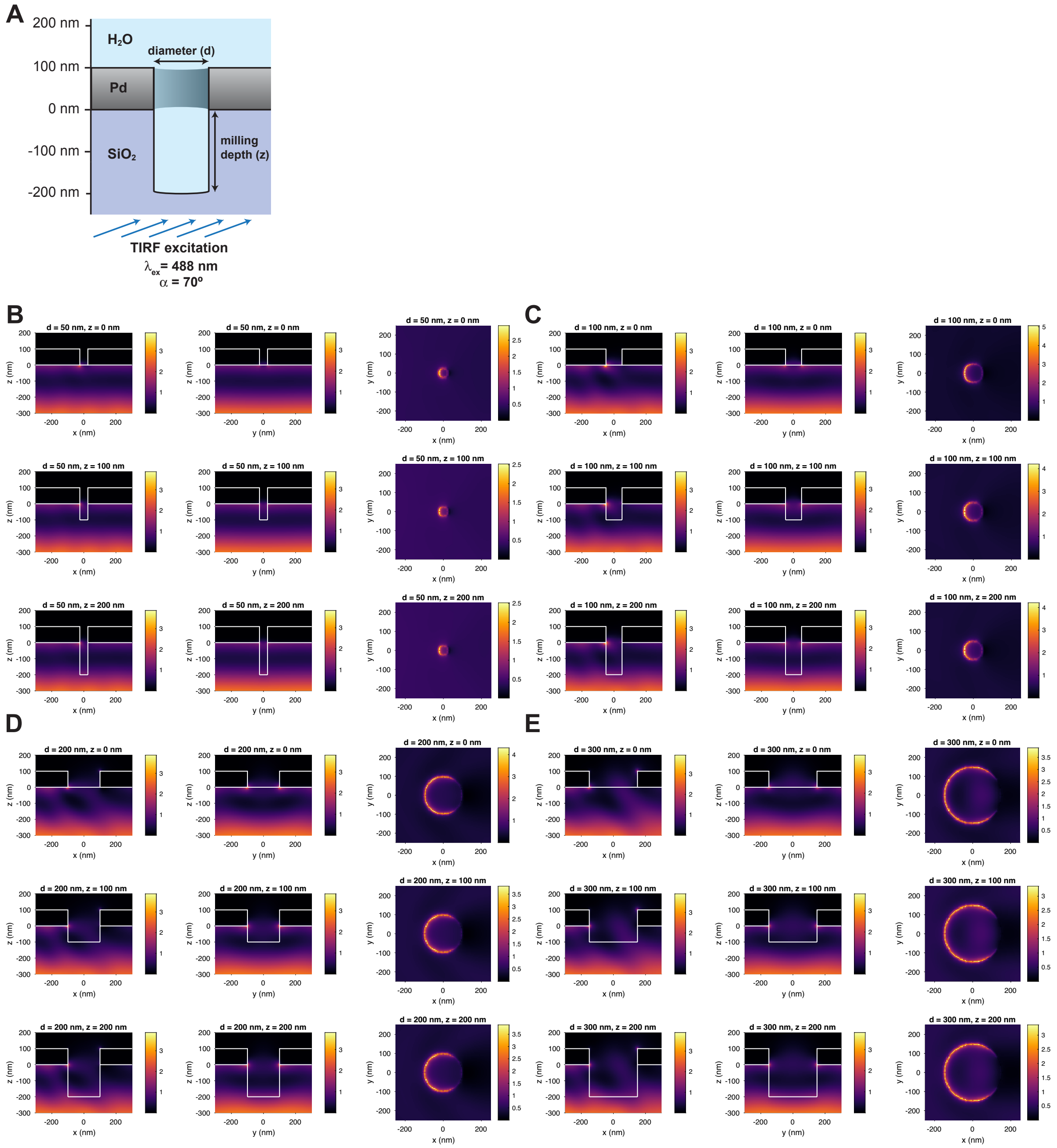

Supplementary Figure 4: **Excitation field intensity distributions from FDTD simulations under TIRF illumination at an angle of 70° at $\lambda_{ex} = 488$ nm. A:** Schematic of the simulation setup. **B-E:** Excitation field intensity distributions $|E|^2$ in V^2/m^2 in the x-z (left), y-z (middle) and x-y plane at the entrance to the ZMW (right) at pore diameters d of 50 nm (B), 100 nm (C), 200 nm (D), and 300 nm (E) and milling depths h of 0 nm (top), 100 nm (middle), and 200 nm (bottom). The electric field is polarized along the x-axis.

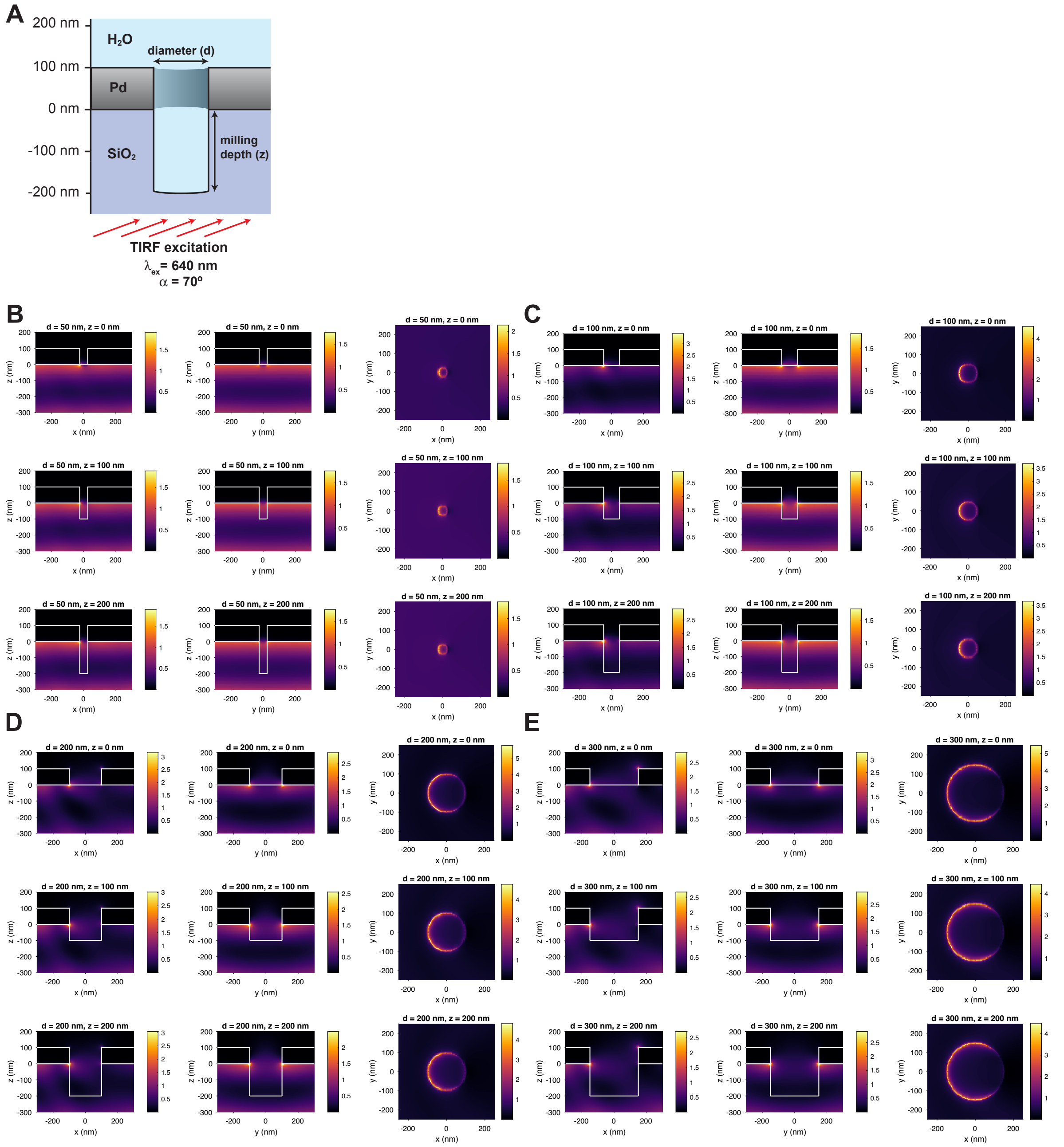

Supplementary Figure 5: **Excitation field intensity distributions from FDTD simulations under TIRF illumination at an angle of 70° at $\lambda_{ex} = 640$ nm. A:** Schematic of the simulation setup. **B-E:** Excitation field intensity distributions $|E|^2$ in V^2/m^2 in the x-z (left), y-z (middle) and x-y plane at the entrance to the ZMW (right) at pore diameters d of 50 nm (B), 100 nm (C), 200 nm (D), and 300 nm (E) and milling depths h of 0 nm (top), 100 nm (middle), and 200 nm (bottom). The electric field is polarized along the x-axis.

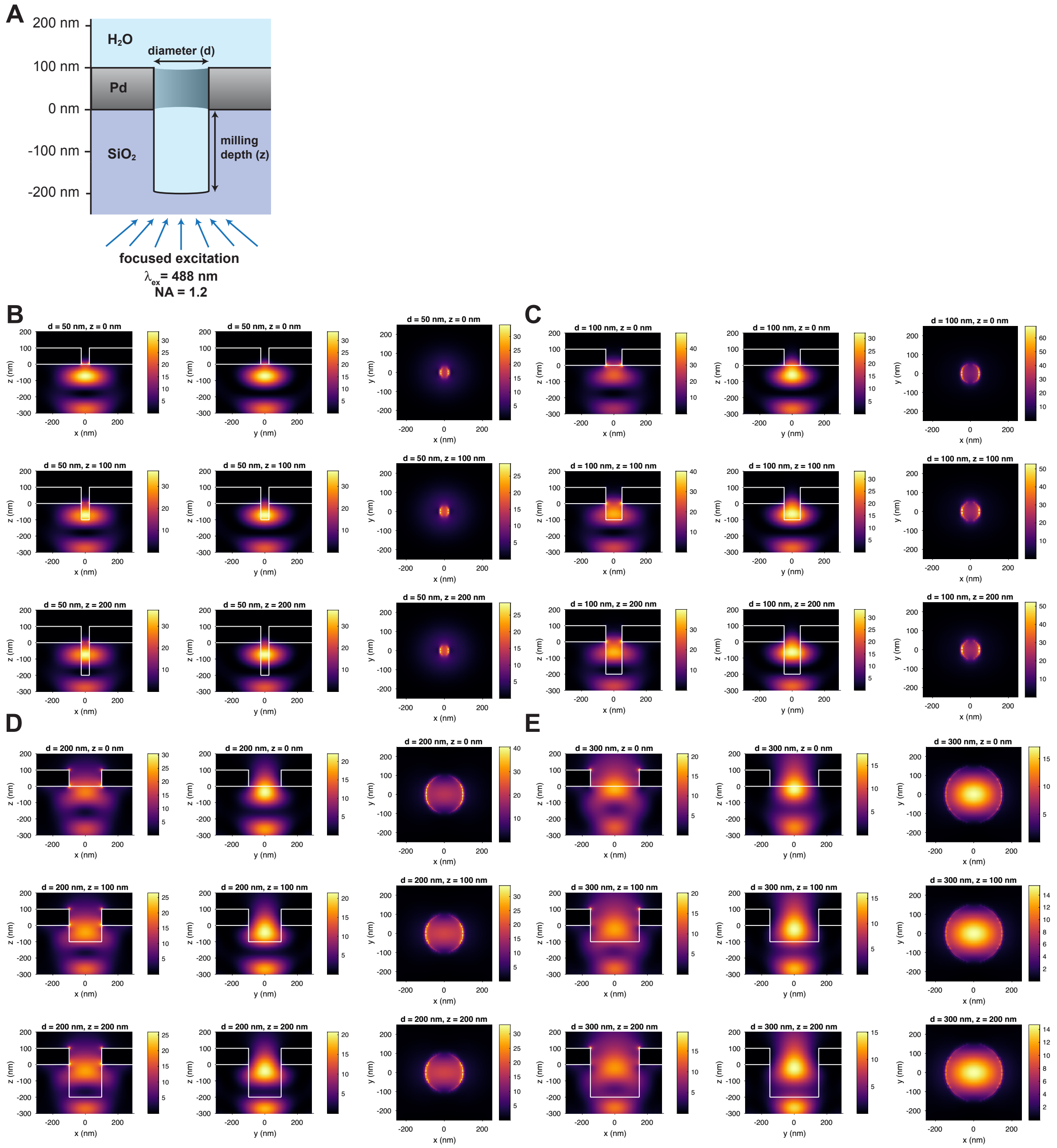

Supplementary Figure 6: **Excitation field intensity distributions from FDTD simulations under excitation by a focused Gaussian beam at NA = 1.2 and a wavelength of $\lambda_{\text{ex}} = 488$ nm.** **A:** Schematic of the simulation setup. **B-E:** Excitation field intensity distributions $|E|^2$ in V^2/m^2 in the x-z (left), y-z (middle) and x-y plane at the entrance to the ZMW (right) at pore diameters d of 50 nm (B), 100 nm (C), 200 nm (D), and 300 nm (E) and milling depths h of 0 nm (top), 100 nm (middle), and 200 nm (bottom). The electric field is polarized along the x-axis.

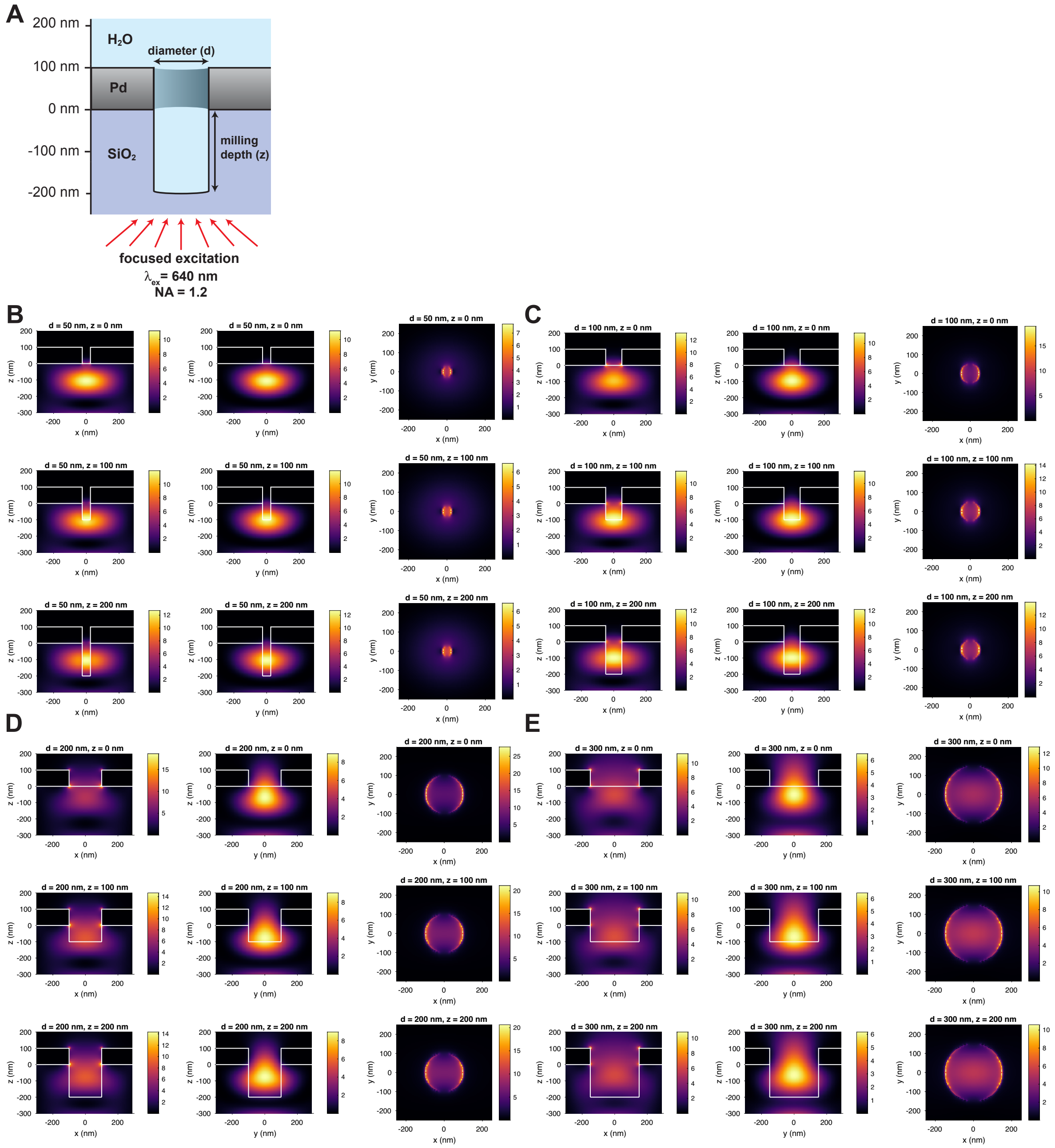

Supplementary Figure 7: **Excitation field intensity distributions obtained from FDTD simulations under excitation by a focused Gaussian beam at NA = 1.2 and $\lambda_{ex} = 640$ nm. A:** Schematic of the simulation setup. **B-E:** Excitation field intensity distributions $|E|^2$ in V^2/m^2 in the x-z (left), y-z (middle) and x-y plane at the entrance to the ZMW (right) at pore diameters d of 50 nm (B), 100 nm (C), 200 nm (D), and 300 nm (E) and milling depths h of 0 nm (top), 100 nm (middle), and 200 nm (bottom). The electric field is polarized along the x-axis.

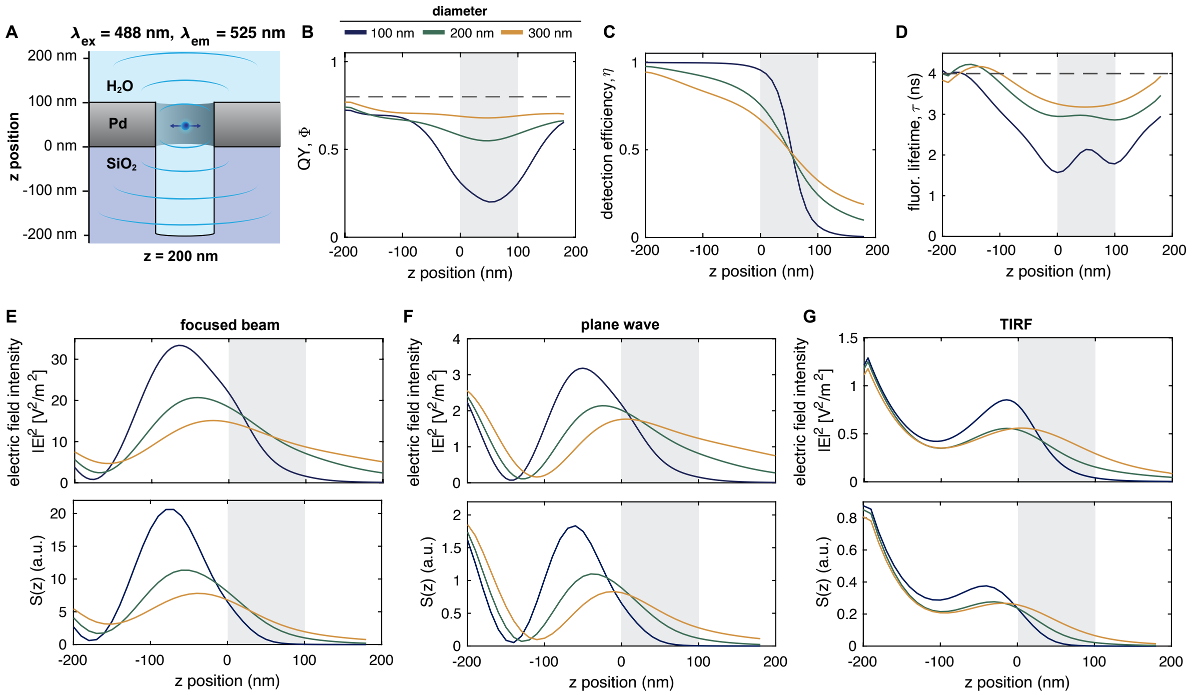

Supplementary Figure 8: **FDTD simulations of fluorescence emission and detected signal from overmilled ZMWs for Alexa488. A:** Schematic of the simulation setup. **B-D:** Computed quantum yield Φ, detection efficiency η, and fluorescence lifetime τ profiles of the dye Alexa488 as a function of the z position. Dashed lines indicate the values in the absence of a ZMW. **E-G:** Z-profiles of the excitation intensity profiles along the central pore axis (top) and the total detected signal S(z) (bottom) under excitation by a focused Gaussian beam (E), plane wave (*i.e.*, widefield) (F) or excitation under TIRF angle (G). The position of the metal membrane is indicated as a gray shaded area.

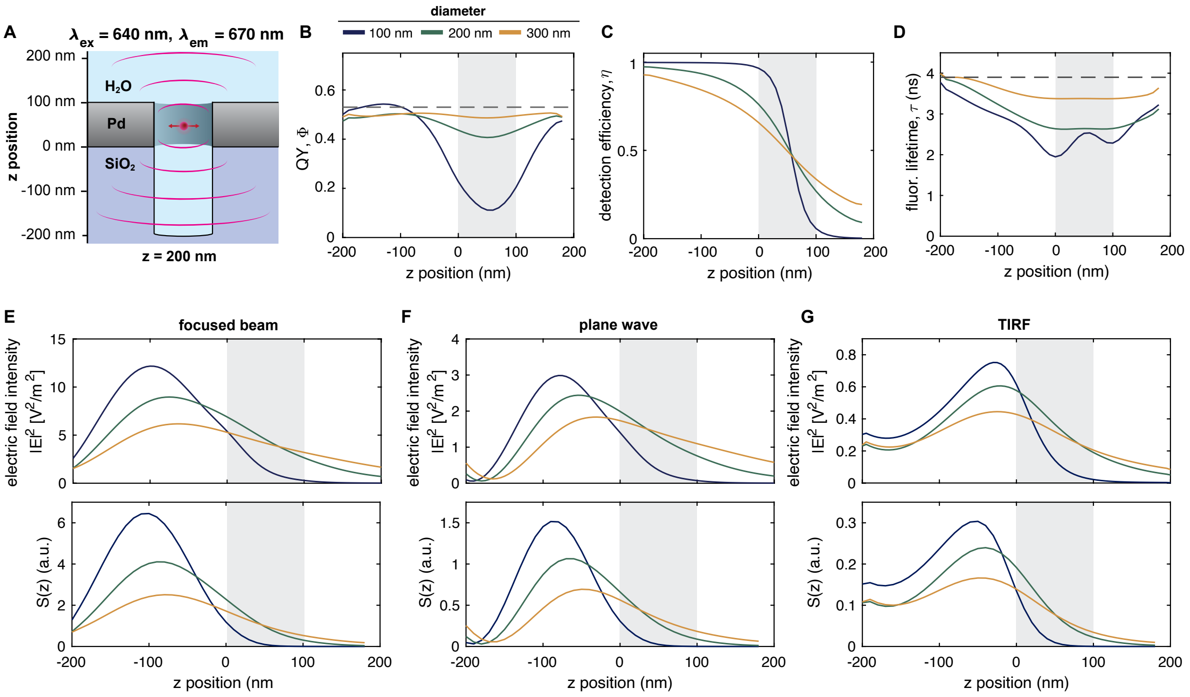

Supplementary Figure 9: **FDTD simulations of fluorescence emission and detected signal from overmilled ZMWs for JFX650. A:** Schematic of the simulation setup. **B-D:** Computed quantum yield Φ, detection efficiency η, and fluorescence lifetime τ profiles of the dye JFX650 as a function of the z position. Dashed lines indicate the values in the absence of a ZMW. **E-G:** Z-profiles of the excitation intensity profiles along the central pore axis (top) and the total detected signal $S(z)$ (bottom) under excitation by a focused Gaussian beam (E), plane wave (*i.e.*, widefield) (F) or excitation under TIRF angle (G). The position of the metal membrane is indicated as a gray shaded area.

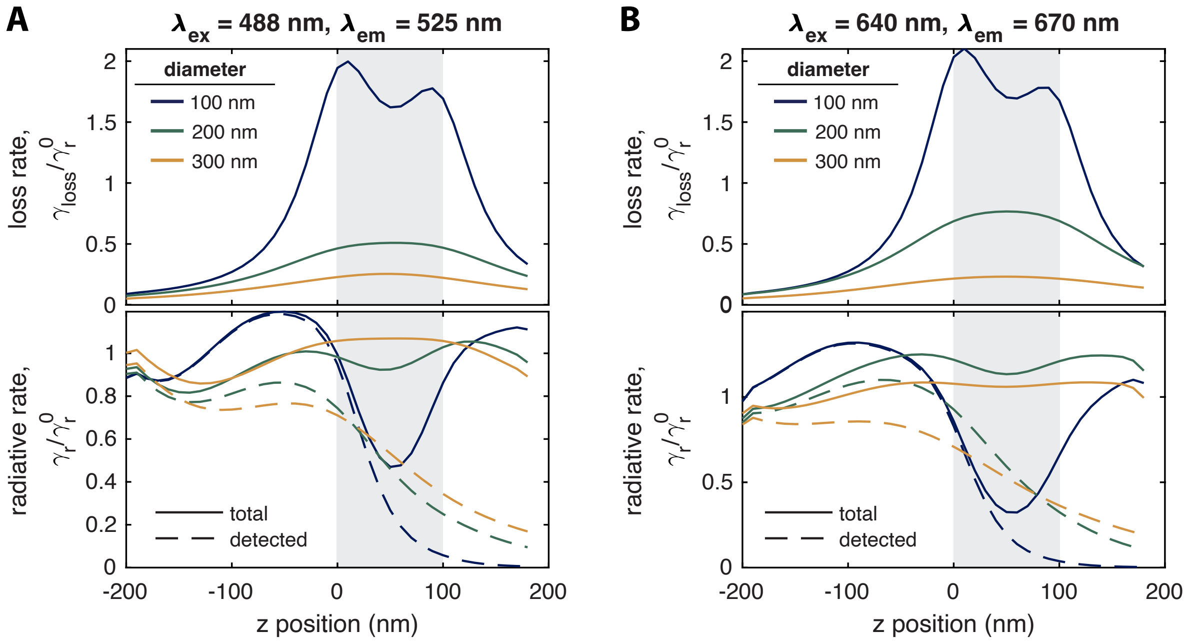

Supplementary Figure 10: **Overview of radiative and non-radiative rates obtained from FDTD simulations of fluorescence emission within overmilled ZMWs.** Shown are the non-radiative loss rate (top) and radiative rate (bottom) in the presence of the metal nanostructure for the dyes Alexa488 (A) and JFX650 (B). The radiative rate towards the detection side is given as a dashed line, from which the detection efficiency is computed. Note that the given rates are normalized to the rates in the absence of the ZMW as described in the methods.

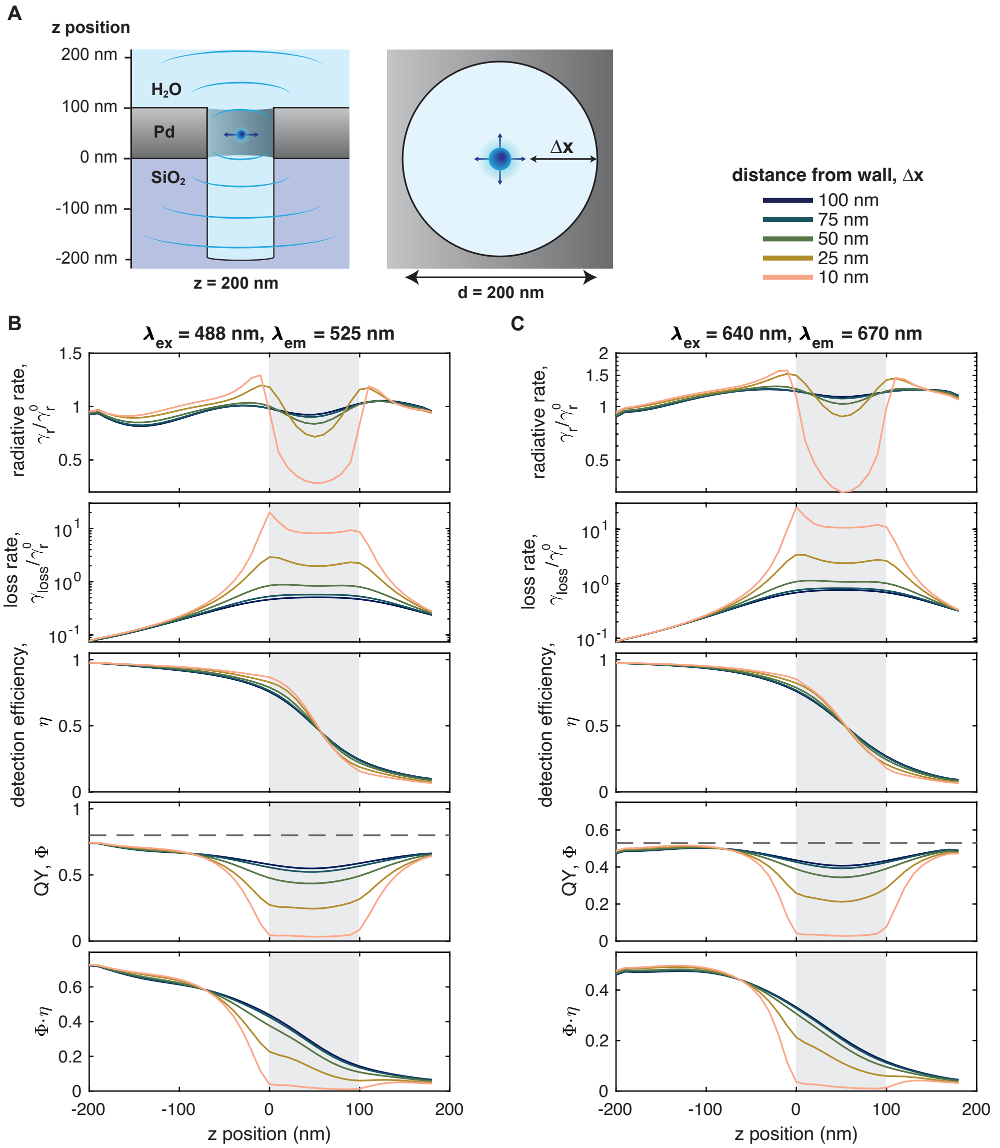

Supplementary Figure 11: **FDTD simulations of dipole emission as a function of the distance to the pore walls. A:** Schematic of the simulation setup. The dipole was placed at varying distances Δx from the pore walls and the emission was monitored as a function of the z-position. **B,C:** Z-profiles of the normalized radiative and loss rates, the detection efficiency η, the quantum yield Φ, and the product of the detection efficiency and quantum yield, $\Phi \cdot \eta$ for Alexa488 (B) and JFX650 (C) at the indicated excitation and emission wavelengths. The quantum yield of the free dye is shown as a dashed line. The dipole emission within the overmilled volume in the glass was found to be approximately independent of the lateral displacement within the pore at distance of ≈ 25 nm away from the ZMW. Within the ZMW, the non-radiative rate is strongly increased as the dipole approaches the pore wall, with significant non-radiative losses occurring at distances below 25 nm that result in a reduction of the quantum yield. Note that the loss rate is given on a log scale. The position of the palladium layer is indicated as a gray shaded area.

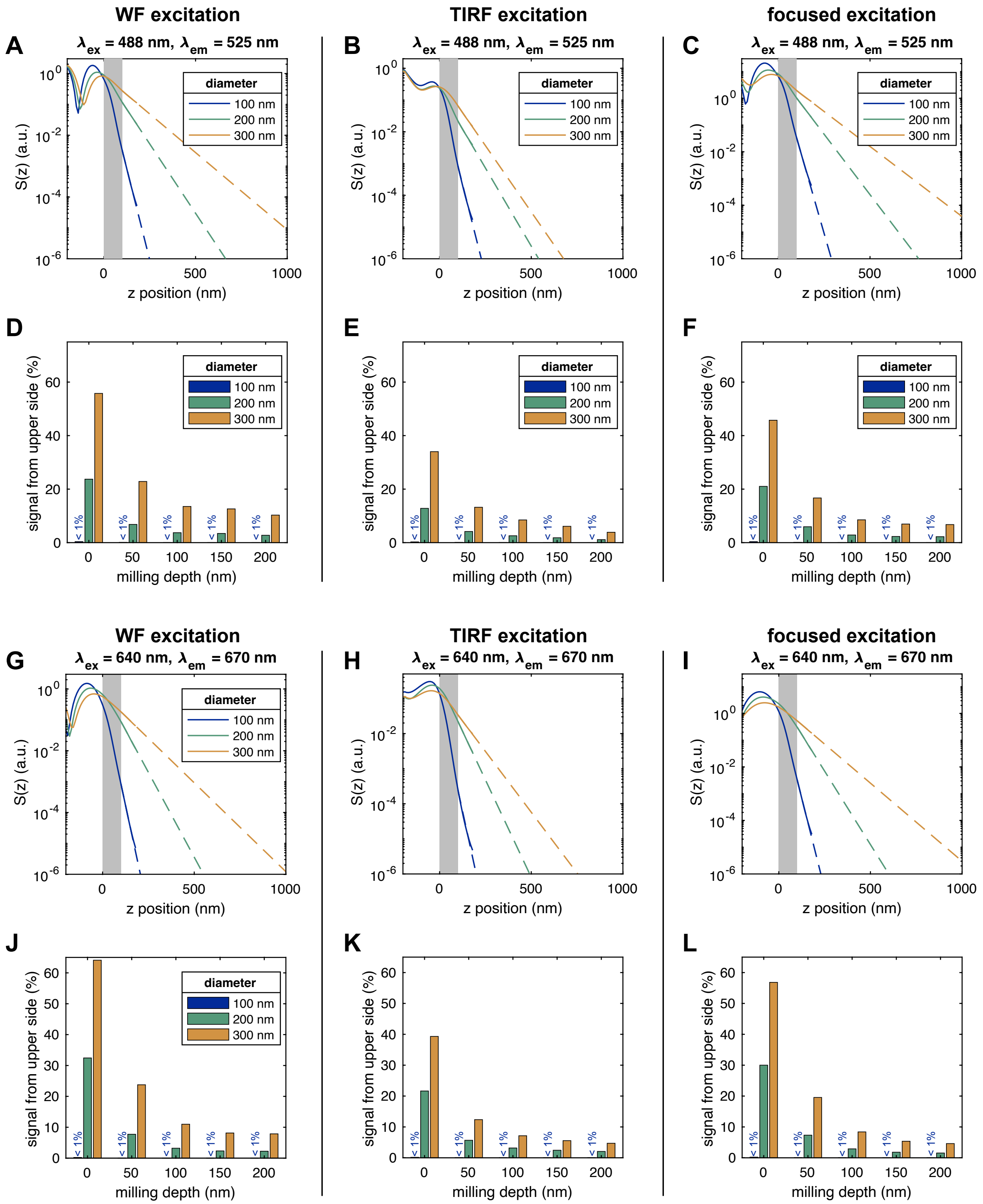

Supplementary Figure 12: **Estimation of background signal from FDTD simulations for the dyes Alexa488 (A-F) and JFX650 (G-L) under the different excitation modes. A-C, G-I:** The detected signal S(z) obtained for widefield (WF), TIRF, and focused excitation (solid lines) is extrapolated by fitting the signal profile in the z-range from 100 nm to 200 nm to an exponential decay (dashed lines). **D-F, J-L:** From the extrapolated signal profiles, the amount of signal detected from the upper side (above a z position of 100 nm) is estimated for different pore diameters and milling depths. The bars for 100 nm are barely visible due to their small height.

Supplementary Figures to Figure 3

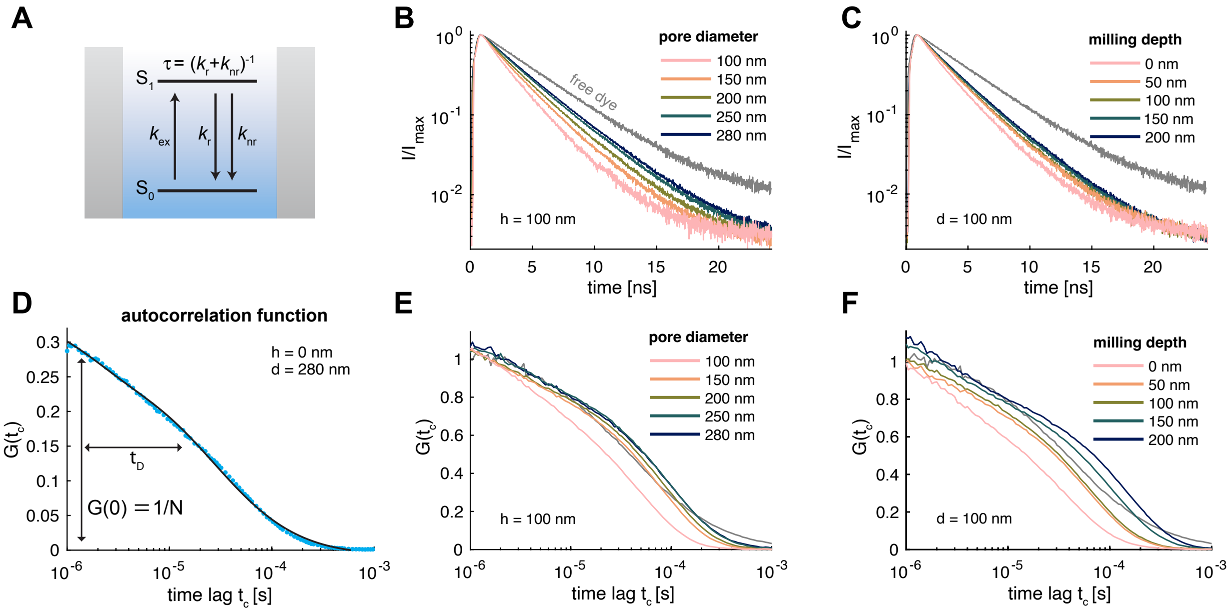

Supplementary Figure 13: **Experimental characterization of photophysics and diffusion within ZMWs. A:** Jablonski scheme of the photophysics in the ZMW. Dyes are radiatively excited from the electronic ground state S_0 to the first excited state S_1 with rate k_{ex}, from where relaxation can occur radiatively (k_r) or non-radiatively (k_{nr}). In the waveguide, all displayed rates change due to excitation field enhancement, plasmonic coupling, and metal-induced quenching. The excited state lifetime reports on the sum of the radiative and non-radiative rates. **B,C:** Fluorescence decays acquired at different pore diameters for a constant milling depth of 100 nm (B) and at different milling depths for a constant pore diameter of 100 nm (C). **D:** Autocorrelation function of the fluorescence time trace shown in Figure 3 C. The FCS analysis informs on the average number of particles (N) and the residence time of molecules within the ZMW (t_D). **E,F:** FCS curves of the data shown in Figure 3 D-F. In B,C,E, and F, the curves for the free dye obtained from a free diffusion experiment are shown in gray.

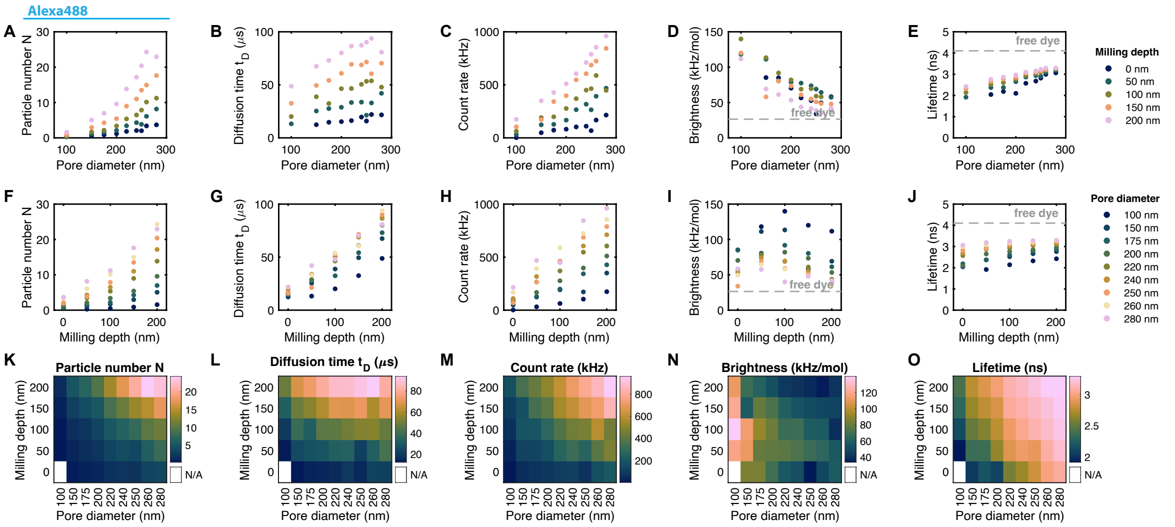

Supplementary Figure 14: **Extracted parameters for the dye Alexa488 in overmilled Pd ZMWs.** Shown are the estimated particle number N, diffusion time t_D , count rate, molecular brightness ε_{ZMW}, and fluorescence lifetime τ as a function of the pore diameter at constant milling depth (A-E), as a function of the milling depth at constant pore diameter (F-J), and as heatmap plots (K-O). The molecular brightness and fluorescence lifetime of the free dye are indicated by gray dashed lines.

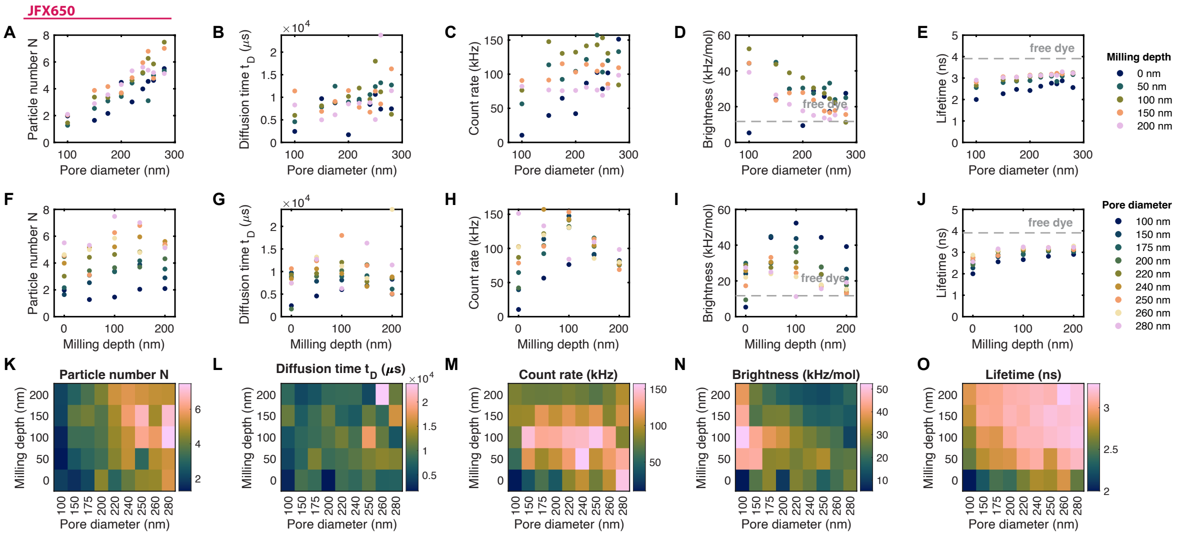

Supplementary Figure 15: **Extracted parameters for the dye JFX650 in overmilled Pd ZMWs.** Shown are the estimated particle number N, diffusion time t_D , count rate, molecular brightness ε_{ZMW}, and fluorescence lifetime τ as a function of the pore diameter at constant milling depth (A-E), as a function of the milling depth at constant pore diameter (F-J), and as heatmap plots (K-O). The molecular brightness and fluorescence lifetime of the free dye are indicated by gray dashed lines. The robustness of the FCS analysis is markedly reduced compared to the Alexa488 dye due to significant sticking of the JFX650 dye to the glass and metal surfaces, as evident from the drastically prolonged diffusion times (B,G,L).

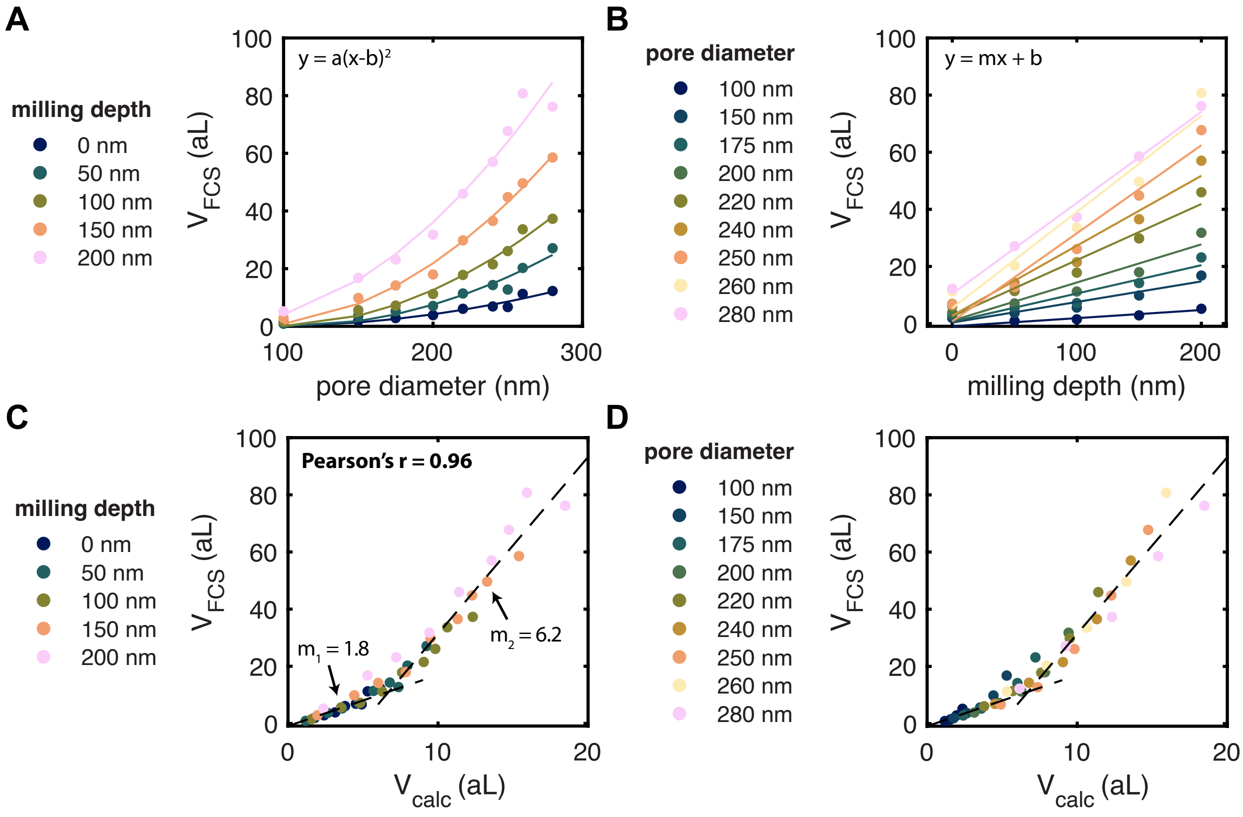

Supplementary Figure 16: **Quantification of the observation volume in overmilled ZMWs. A,B:** Estimated effective volumes V_{FCS} as a function of the pore diameter (A) and milling depth (B). The effective volume V_{FCS} was estimated from the particle number N determined from FCS as $V_{\mathrm{FCS}} = N/c$, where c is the concentration of the dye ($c = 500$ nM). Note that the effective volume in FCS corresponds to a hypothetical volume with constant signal that contains N particles[S1,S2]. The effective volume shows a quadratic scaling with the pore diameter and linear scaling with the milling depth, as expected for the approximately cylindrical volume of the overmilled ZMWs. **C,D:** Comparison of the calculated volume of the overmilled aperture in the glass, V_{calc}, and the experimentally determined effective volume, V_{FCS} , color coded by milling depth (C) and pore diameter (D). V_{calc} is given by $V_{\mathrm{calc}} = (\pi/4)d^2(z + l)$, where d is the diameter, z the overmilling depth, and l is the thickness of the Pd layer ($l = 100$ nm). An excellent correlation is observed between the two quantities (Pearson's correlation coefficient $r = 0.96$), however the measured volumes V_{FCS} are consistently overestimated by a factor of ≈ 2 for small pores ($V_{\mathrm{calc}} \leq 5$ aL) and ≈ 6 for large pores ($V_{\mathrm{calc}} \geq 10$ aL). Note that this overestimation is also present at small pore diameters where only little signal is detected from within the ZMW. Similar overestimation of the particle numbers within ZMW by FCS have previously been reported and attributed to a contribution of constant signal from many dim particles outside of the ZMW[S2–S5].

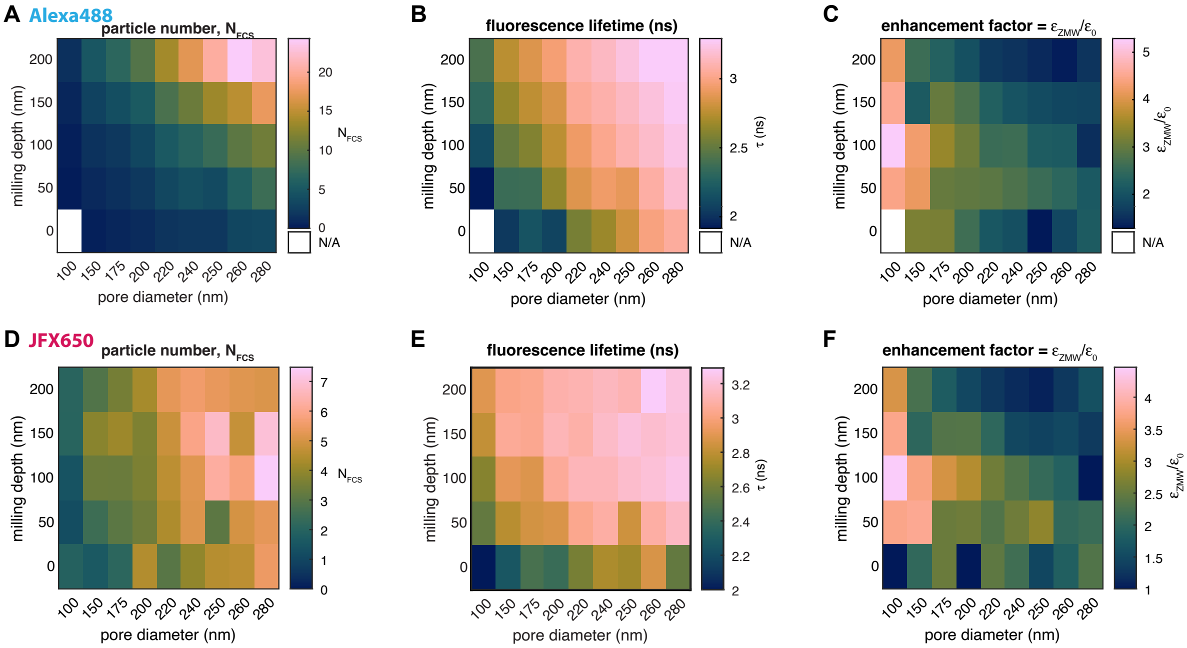

Supplementary Figure 17: **Comparison of extracted parameters for the dyes Alexa488 and JFX650 in ZMW.** The heatmap plots show the average number of particles in the observation volume N, fluorescence lifetime τ, and signal enhancement factor for the dyes Alexa488 (A-C) and JFX650 (D-F). The enhancement factor is defined as the ratio of the counts per molecule in the ZMW compared to free diffusion, $\varepsilon_{\text{ZMW}}/\varepsilon_0$. Data marked as N/A could not be quantified due to insufficient signal. The trends visible for Alexa488 are not as clear with JFX650 due to non-specific sticking interactions of the fluorophore with the surface, as evidenced by the increased diffusion time (see Figure 3—Supplementary Figure 15).

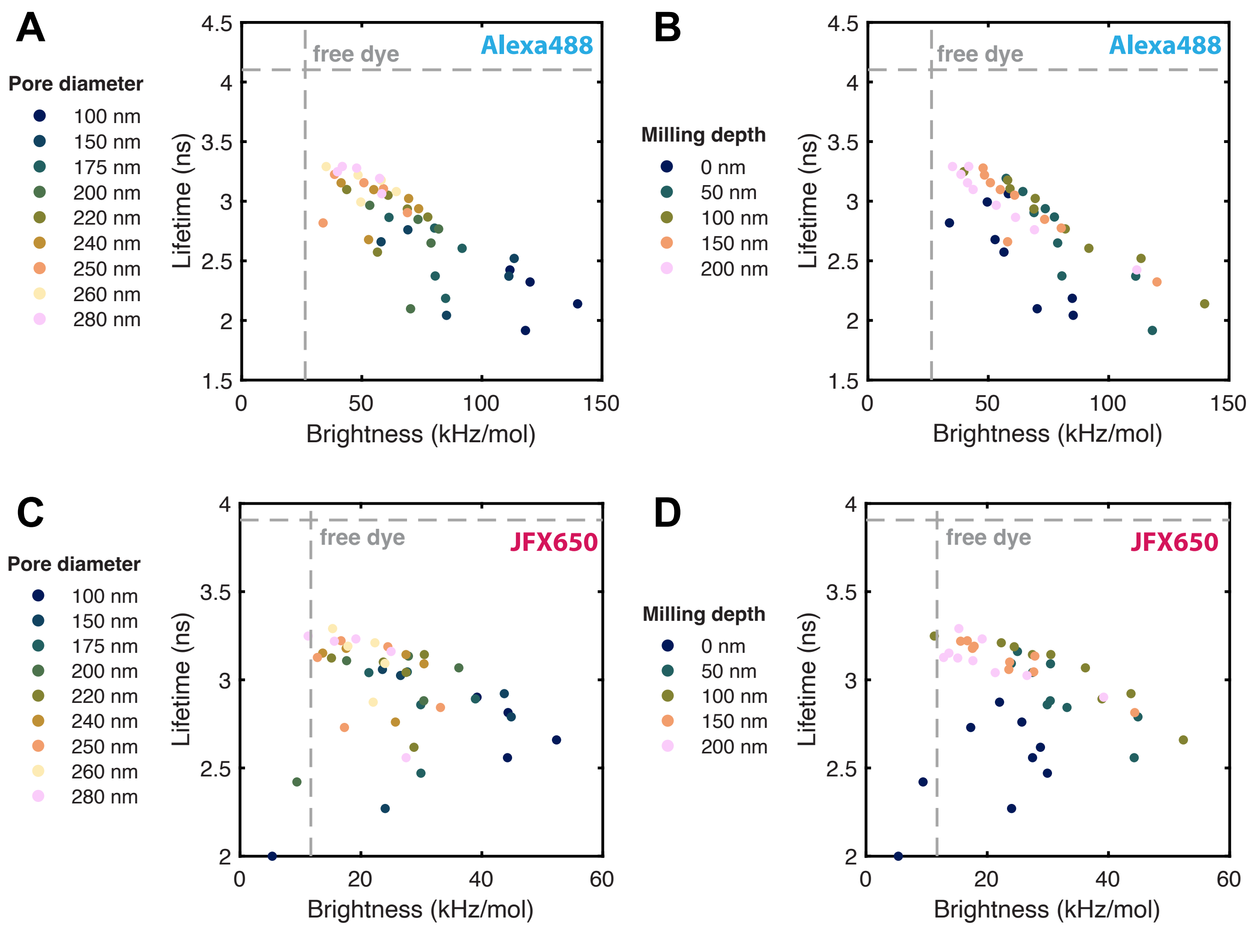

Supplementary Figure 18: **Correlation between fluorescence lifetime and molecular brightness in ZMW for Alexa488 (A,B) and JFX650 (C,D).** The data is color coded either by pore diameter (A,C) or milling depth (B,D). The fluorescence lifetime is given by the inverse of the excited state decay rate. A reduction of the lifetime thus indicates the enhancement of the radiative and/or non-radiative relaxation rates due to the ZMW. A negative correlation is observed, where a lower lifetime corresponds with an increased molecular brightness.

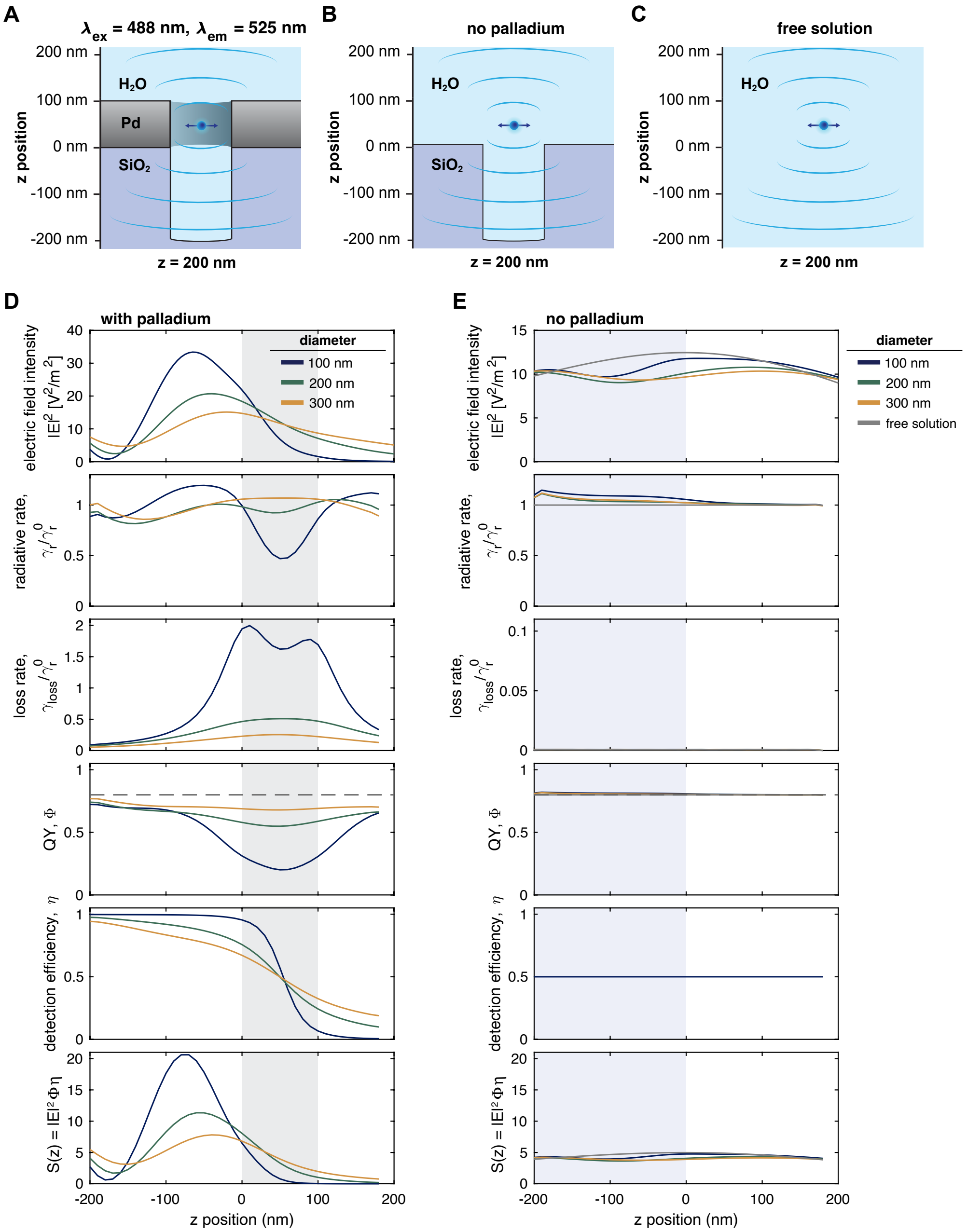

Supplementary Figure 19: **FDTD simulations of excitation field and fluorescence emission in the absence of a Pd layer. A,B:** Schematic of the simulation setup with (A), without (B) the Pd layer and for the free solution case (C). **D,E:** Computed excitation field intensity $|E^2|$, normalized radiative rate γ_r/γ_r^0, normalized loss rate $\gamma_{\text{loss}}/\gamma_r^0$ quantum yield Φ, detection efficiency η, and total fluorescence signal S(z) as a function of the z position in the presence (D) and absence (E) of the Pd layer. In D, the position of the metal membrane is indicated as a gray shaded area. In E, the position of the SiO_2 layer is indicated as a blue shaded area (except for the free diffusion case). The detection efficiency was set to 0.5 in the absence of the Pd layer. A small radiative rate enhancement arises even in the absence of the Pd layer when the dipole is placed in the SiO_2 nanocavity due to the Purcell effect[S6].

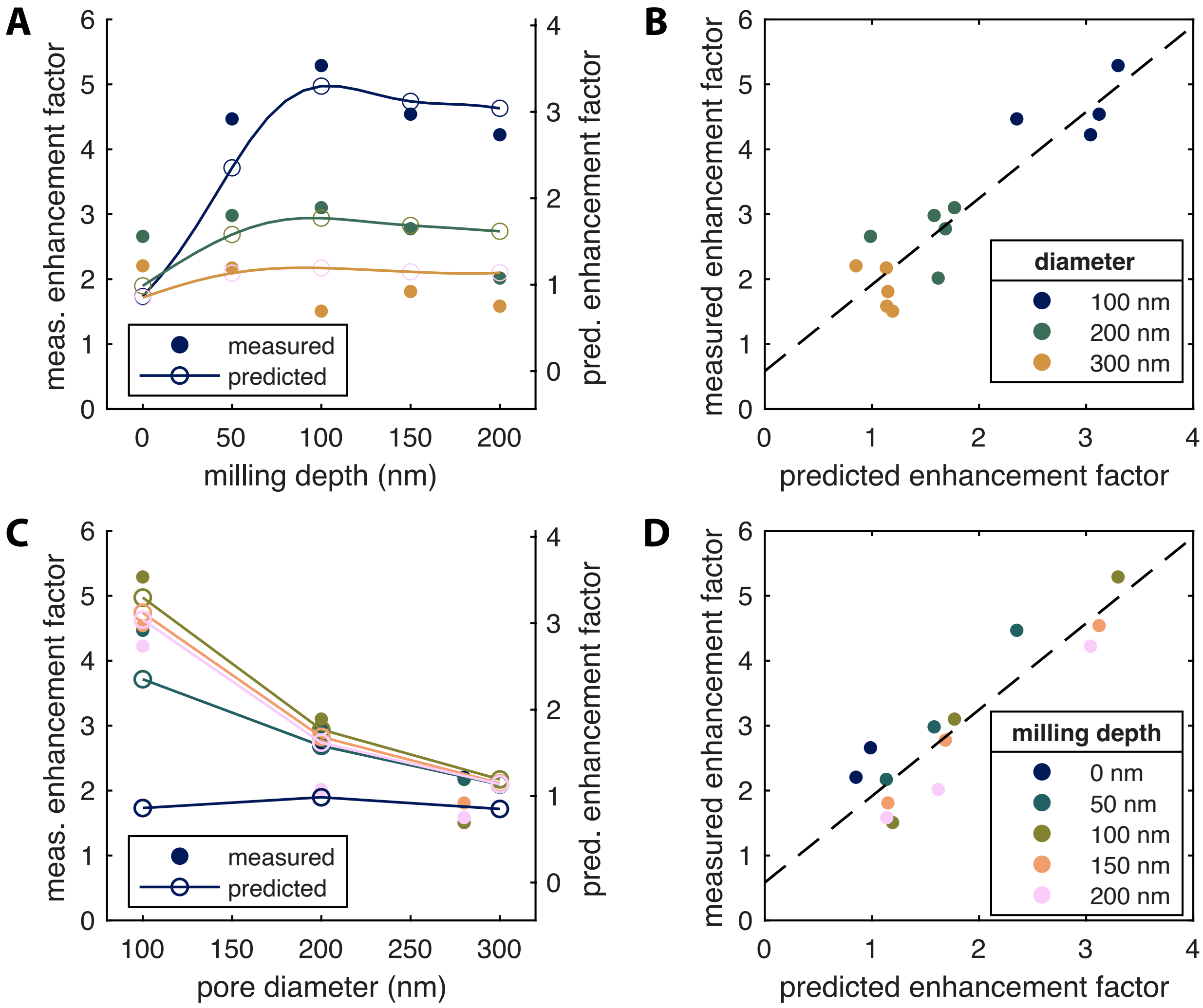

Supplementary Figure 20: **Comparison of experimental and predicted enhancement factors in overmilled Pd ZMWs. A,C:** Measured and predicted enhancement factors as a function of the milling depths (A) or pore diameter (C). The scaling of the two y-axes was adjusted according to the results of the linear regression between measured and predicted enhancement factors. **B,D:** Plots of the measured versus the predicted enhancement factors, color coded by milling depth (B) or pore diameter (D). The solid line is a linear fit given by $y = 1.33x + 0.58$. The Pearson correlation coefficient is $r = 0.92$.

Supplementary Figures to Figure 4

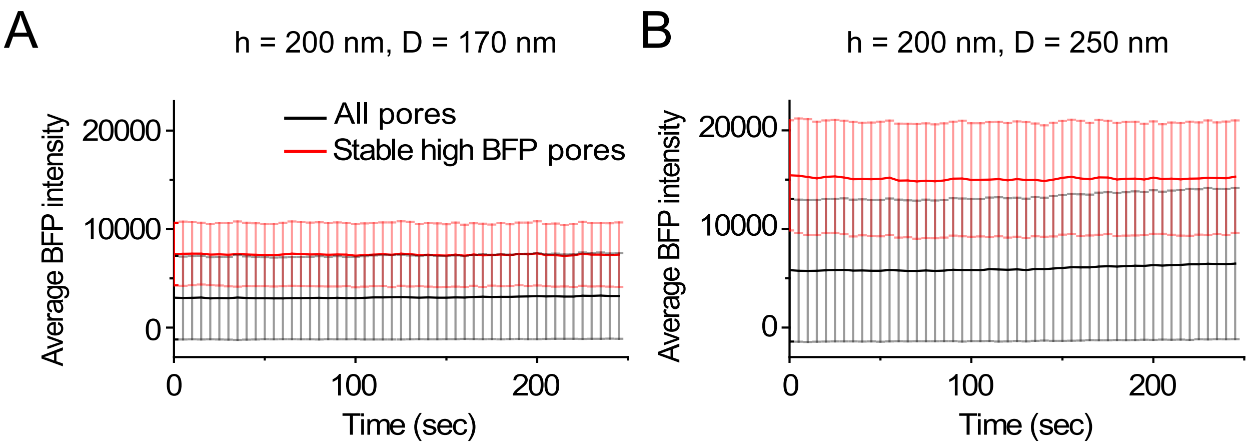

Supplementary Figure 21: **Stability of the BFP signal during the experiments.** (A) Time dependence of the average BFP intensity across for all pores (black) and for pores showing high BFP signal (red). The milling depth was 200 nm and the diameter was 170 nm. (B) Same as panel A for a pore diameter of 250 nm. Error bars represent the standard deviation of the signal. No photobleaching was observed over the course of the experiment.

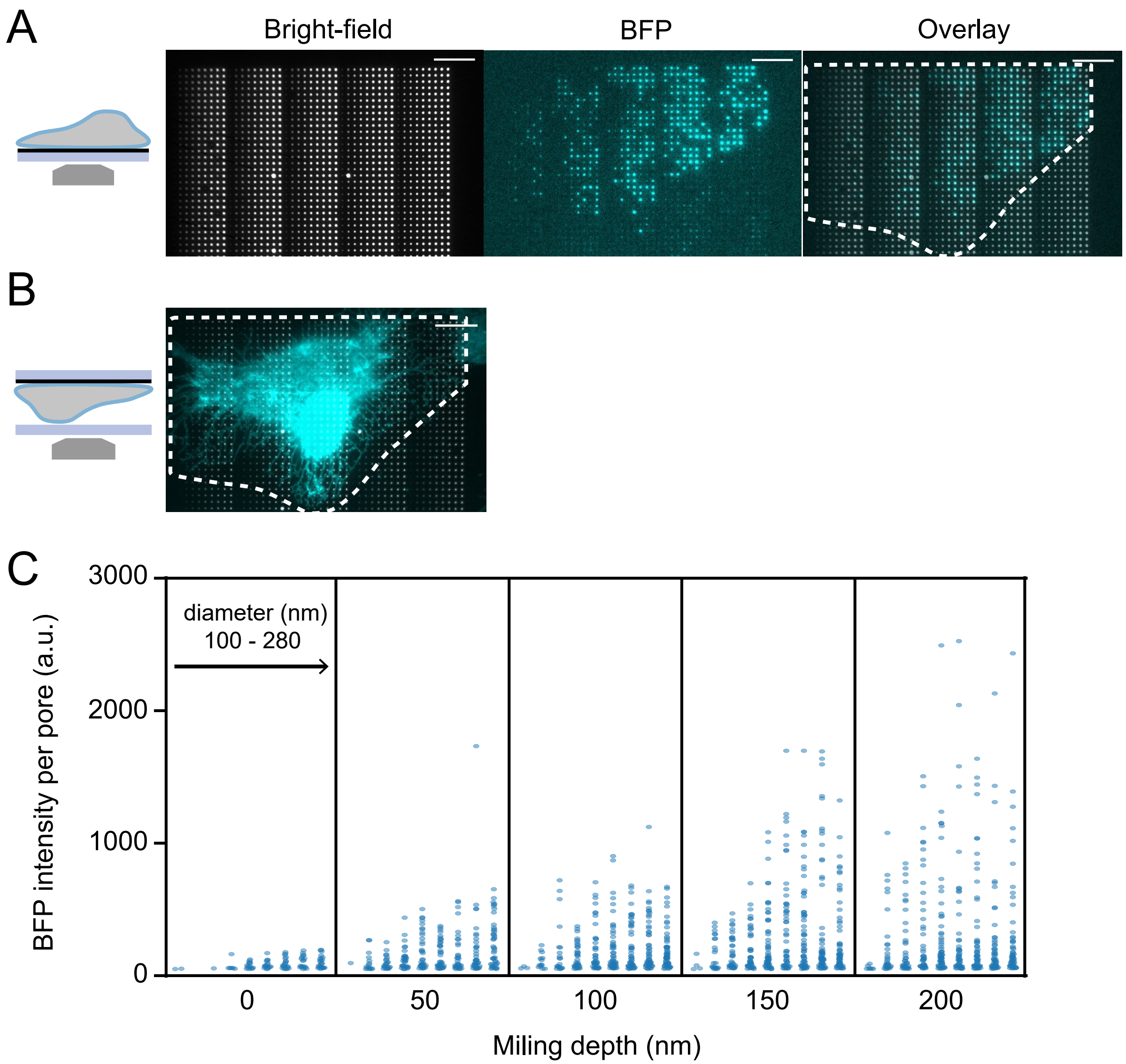

Supplementary Figure 22: **Correlated imaging of flipped coverslips confirms the colocalization of pores with BFP signal with the presence of cells on the ZMW array.** A: From left to right, bright-field, BFP fluorescence, and composite images of the experiment shown in Figure 4 A, depicting cells attached to the coverslip. B: Overlay of bright-field and BFP fluorescence images obtained from the same field of view as in (A) after flipping the orientation of the coverslip, where cells attached to the Pd surface are not imaged through the ZMWs but from the top side. Note that the image is mirrored to visualize cells in the same orientation as in (A). Scale bars: 10 µm. C: BFP fluorescence intensities of individual pores with varying sizes. Each dot represents a single pore. The total number of analyzed pores was 1890.

Supplementary Figures to Figure 5

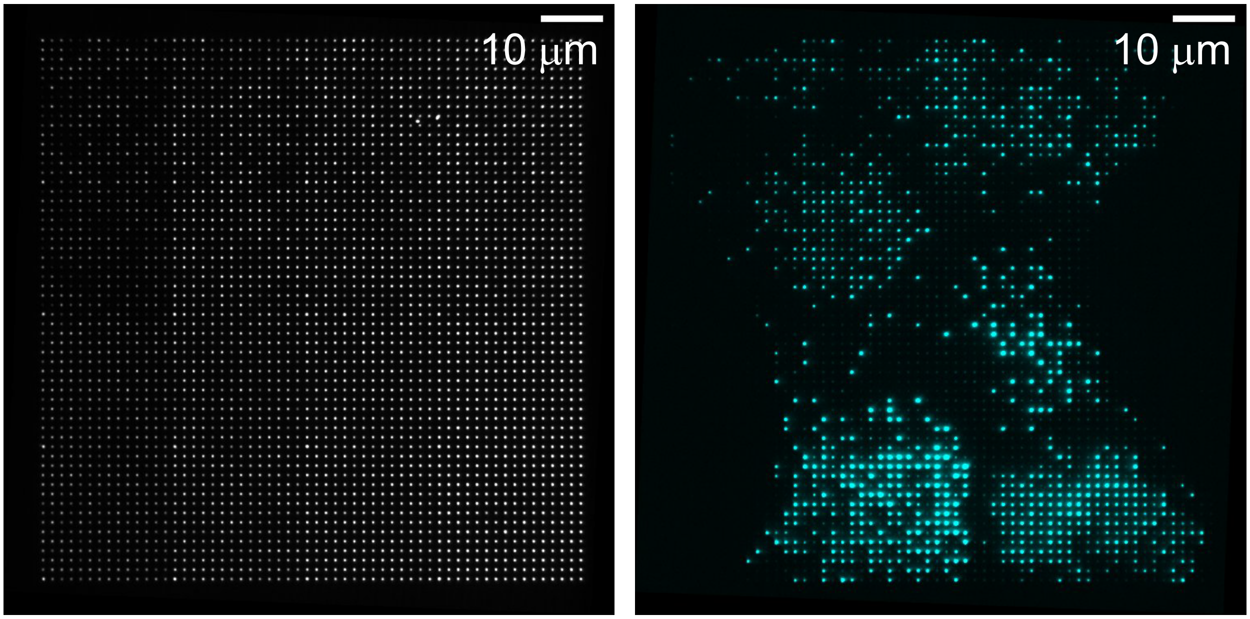

Supplementary Figure 23: **Brightfield and BFP-fluorescence images of array version 2** Orientation is the same as in Figure 1—Supplementary Figure 1 F. The brightfield image (left) shows the location of pores and the BFP-fluorescence image (right) shows their occupation.

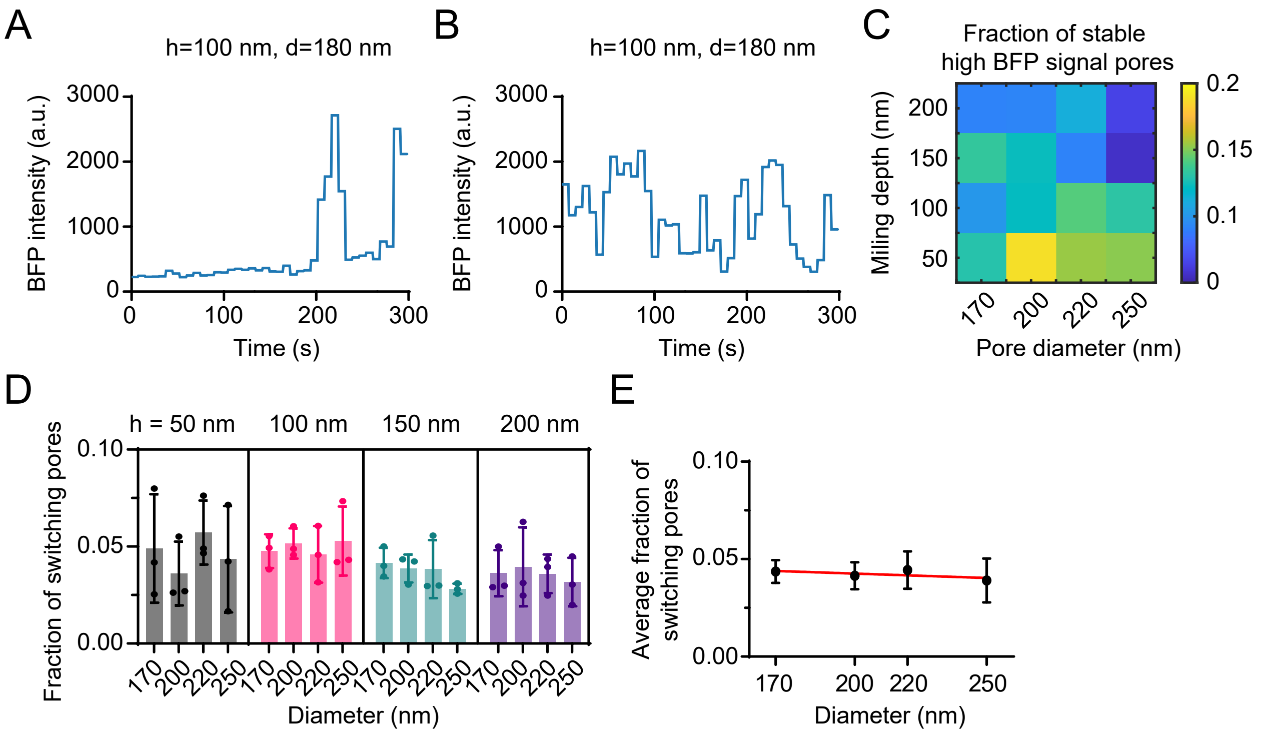

Supplementary Figure 24: **BFP fluorescence intensity switching between high and low signal levels.** **A,B:** Representative examples of BFP fluorescence intensity time traces for individual pores where the intensity switched between the high and low intensity levels. **C:** Percentage of pores with high stable BFP signal for the duration of the movie (300 s). The total number of pores analyzed is 30907, with approximately 1900 pores for each pore size. **D:** Fraction of pores exhibiting switching between different BFP intensity levels as a function of pore size. Given are the mean and standard deviation from three independent experiments. Each dot represents an individual experiment. **E:** Average fraction of pores showing switching behavior versus diameter. No significant dependence of switching on diameter and milling depth is evident.

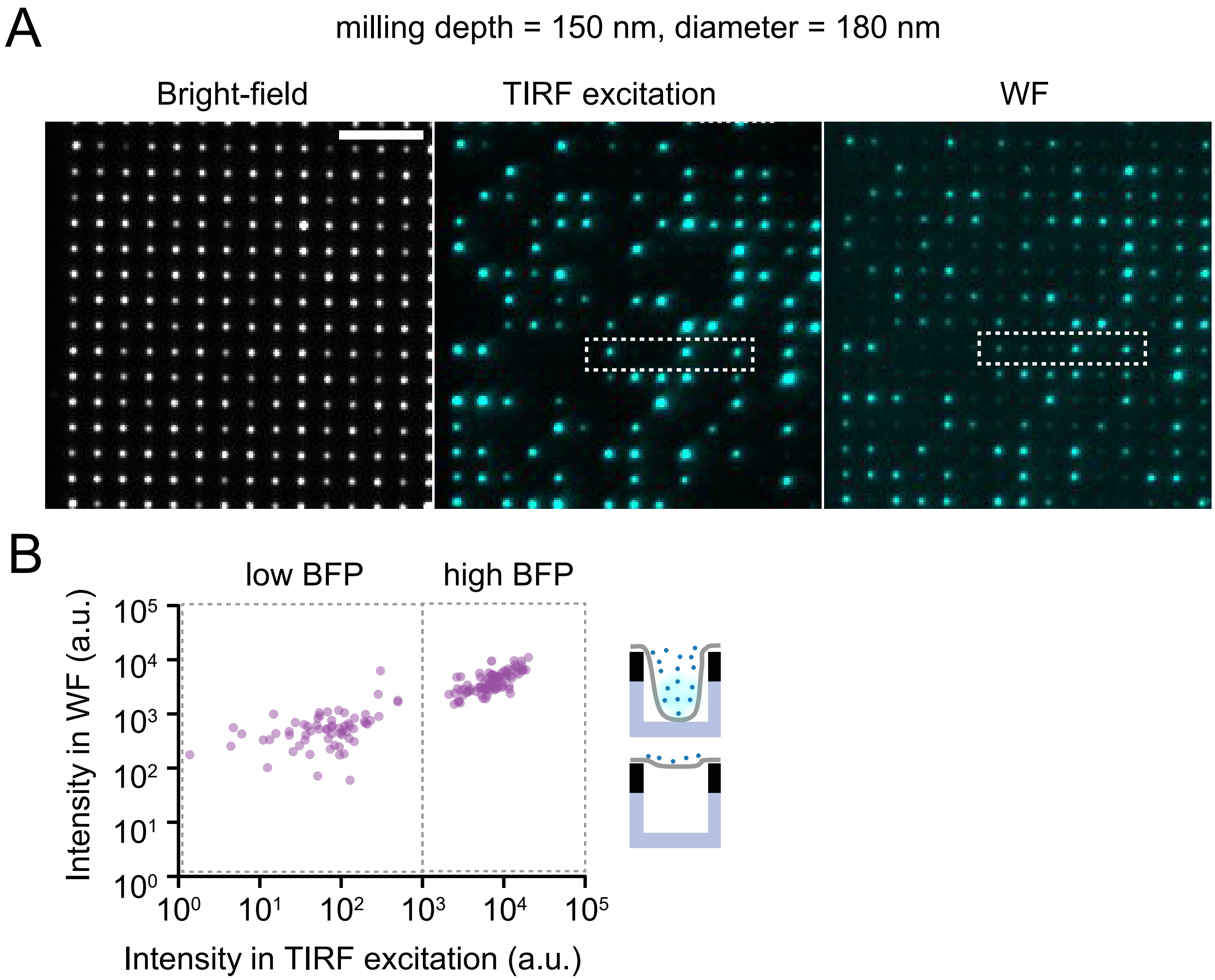

Supplementary Figure 25: **TIRF illumination vs. widefield illumination. A:** Bright-field image (left), and BFP fluorescence images acquired with either TIRF illumination (center) or WF illumination (right) of nanopores with milling depth $h = 150\,\mathrm{nm}$ and diameter $d = 180\,\mathrm{nm}$. Scale bar, 5 µm. The boxes indicate the location of the zoom-in shown in Figure 5 G. **B:** Scatter plot of BFP intensities in individual pores for TIRF and widefield illumination, with each dot representing a single pore. Note that the two populations can be distinguished much more readily when using TIRF excitation compared to widefield illumination. The number of pores analyzed is 150.

Supplementary Figures to Figure 6

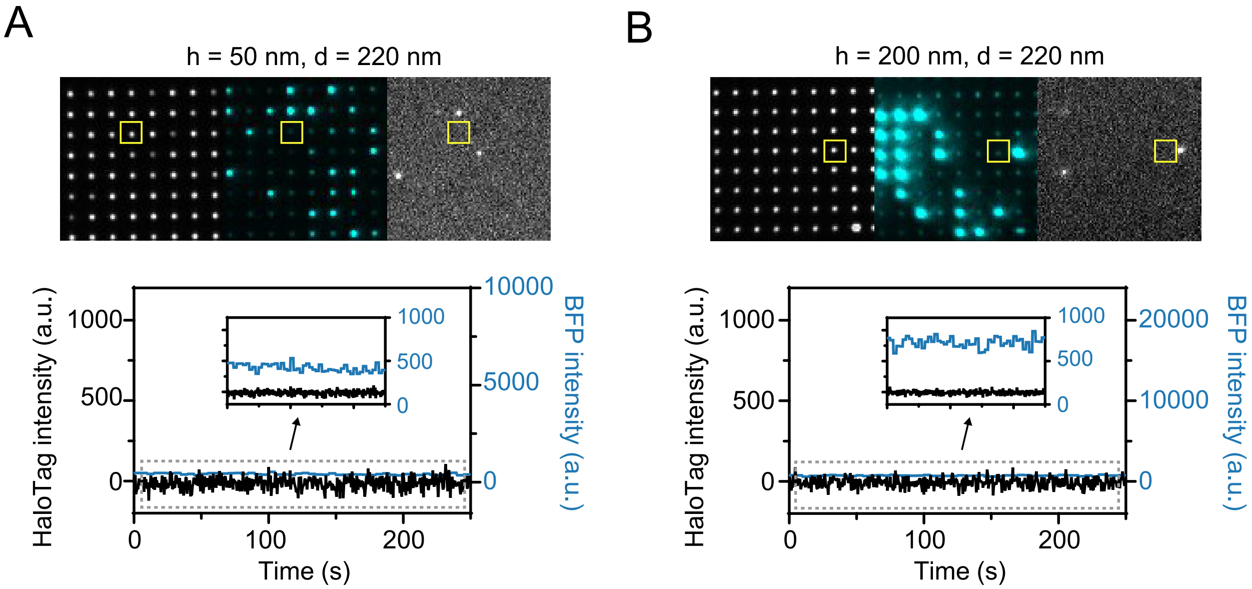

Supplementary Figure 26: **BFP and JFX650-HaloTag signal of pores showing low BFP signal.** Bright-field, BFP, and JFX650-HaloTag signal from representative pores showing only low BFP signal with milling depths of 50 nm (left) or 200 nm (right). A fluorescence time trace of a single pore is shown for each condition. Insets provide a zoom-in on the grey box. While a low signal from BFP is observed, no single molecule events of the red fluorophore could be detected. Time interval, 5 s for BFP, 500 ms for JFX650-Halo

References

(S1) Xu, C.; Webb, W. W. In *Topics in Fluorescence Spectroscopy: Volume 5: Nonlinear and Two-Photon-Induced Fluorescence*; Lakowicz, J. R., Ed.; Springer US: Boston, MA, 2002; Chapter 11, pp 471–540.

(S2) Levene, M. J.; Korlach, J.; Turner, S. W.; Foquet, M.; Craighead, H. G.; Webb, W. W. Zero-mode waveguides for single-molecule analysis at high concentrations. *science* **2003**, *299*, 682–686.

(S3) Rigneault, H.; Capoulade, J.; Dintinger, J.; Wenger, J.; Bonod, N.; Popov, E.; Ebbesen, T. W.; Lenne, P.-F. Enhancement of Single-Molecule Fluorescence Detection in Subwavelength Apertures. *Phys. Rev. Lett.* **2005**, *95*, 117401.

(S4) Lenne, P.-F.; Rigneault, H.; Marguet, D.; Wenger, J. Fluorescence fluctuations analysis in nanoapertures: physical concepts and biological applications. *Histochemistry and Cell Biology* **2008**, *130*, 795.

(S5) Wu, M.; Liu, W.; Hu, J.; Zhong, Z.; Rujiralai, T.; Zhou, L.; Cai, X.; Ma, J. Fluorescence enhancement in an over-etched gold zero-mode waveguide. *Optics express* **2019**, *27 13*, 19002–19018.

(S6) Purcell, E. M. Spontaneous Emission Probabilities at Radio Frequencies. *Phys. Rev. Lett.* **1946**, *69*, 839–839.
